# Plant–plant interactions determine natural restoration of plant biodiversity over time, in a degraded mined land

**DOI:** 10.1002/ece3.8878

**Published:** 2022-04-30

**Authors:** Maral Bashirzadeh, Richard P. Shefferson, Mohammad Farzam

**Affiliations:** ^1^ 48440 Department of Range and Watershed Management Faculty of Natural Resources and Environment Ferdowsi University of Mashhad Mashhad Iran; ^2^ Organization for Programs on Environmental Sciences Faculty of Arts & Sciences University of Tokyo Tokyo Japan

**Keywords:** functional diversity, life form, mining limestone, nurse plant, phylogenetic diversity, plant biodiversity, restoration

## Abstract

Restoration of degraded environments is essential to mitigate adverse impacts of human activities on ecosystems. Plant–plant interactions may provide effective means for restoring degraded arid lands, but little is understood about these impacts. In this regard, we analyzed the effects of two dominant nurse plants (i.e., *Artemisia sieberi* and *Stipa arabica*) on taxonomic, functional, and phylogenetic diversity across different ages of land abandonment (i.e., control, recent, and old ages) in a limestone mine site in Iran. In addition, we considered two spatial scales: i) the plot scale (i.e., under 1m2 plots) and ii) the vegetation‐patch scale (i.e., under the canopies of nurse plants), to assess nurse plant effects, land abandonment ages, and their relative importance on biodiversity facets by performing Kruskal–Wallis H test and variation partitioning analysis. Our results indicated an increase in taxonomic, functional, and phylogenetic diversity at the plot scale, when considering the presence of nurse plants under old ages of land abandonment. Such significant differences were consistent with the positive effects of *Artemisia* patches on taxonomic diversity and *Stipa* patches on functional and phylogenetic diversity. In addition, we found a larger contribution from nurse plants than land abandonment age on biodiversity variation at both spatial scales studied. Therefore, these results indicate the importance of plant–plant interactions in restoring vegetation, with their effects on the presence of beneficiary species and their functional and phylogenetic relatedness depending on the nurse life forms under the stress‐gradient hypothesis.

## Graphical abstract

We analyzed the effects of nurse plants on biodiversity facets at community and vegetation‐patch scales across different ages of land abandonment in Cement‐Shargh plant in Iran. Our results indicated significant variations in biodiversity depending on presence the nurse plants. Such significant differences in biodiversity were consistent with positive effects of nurse shrubs on taxonomic diversity and grasses on functional and phylogenetic diversity at vegetation‐patch scale.

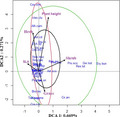

## 1 INTRODUCTION

Mining limestone for cement is a major industrial activity in at least 15 countries (Mucina et al., 2019; Pratiwi et al., 2021). Today, the number of ecosystems destroyed by these mining activities is increasing, and many studies have shown negative impacts of the presence of post‐mining sites on ecosystem health (Palmer et al., 2010). Therefore, the restoration of degraded sites is essential to mitigate the adverse impacts of mining on the ecosystem (Khater & Arnaud, 2007; Mucina et al., 2019). Restoration is a long process, as affected ecosystems have lost their biodiversity and most of their ecosystem functions and services (Sandell Festin et al., 2019). Ecologists preferably emphasize the re‐establishment or increase of biodiversity as a goal of restoration. Biodiversity is typically associated with the increase in ecosystem functions or ecosystem services (Mucina et al., 2019; Pratiwi et al., 2021; Rey Benayas et al., 2008). In this regard, assessing biodiversity and ecosystem services in post‐mining sites has received wide attention among restoration scientists (Pitz et al., 2015). However, there is still little understanding of the long‐term changes in plant biodiversity and appropriate approaches for enhancing biodiversity in areas degraded after quarrying of limestone and other minerals.

Several approaches and measures are typically used to bring back the lost ecosystems that have suffered from mining (Adesipo et al., 2021; Bradshaw, 2000; Giannini et al., 2016). In recent decades, some conventional techniques such as waste removal, dam building, and on‐site containment by sealing have usually been applied for the restoration of mining‐affected lands (European Commission, 2009). However, these methods are criticized for being cost‐effective, time‐consuming and unacceptable to the general public, and impossible to apply at large scales due to geo‐structural and environmental restrictions (Adesipo et al., 2021; Conesa & Schulin, 2010; Tordof et al., 2000). Therefore, restoration ecologists recently suggested plant‐based techniques to restore degraded ecosystems in a cost‐effective and environmentally friendly manner compared with other approaches based on some recent conceptual models hypothesizing plant facilitation as an interaction exploitable in restoration (Cuevas et al., 2013; Navarro‐Cano et al., 2017; Ren et al., 2008). In such restoration projects, the key target is usually the re‐establishment of the plant community by foundation species such as nurse plants, which ameliorate severe conditions for the recruitment and survival of plant species (Giannini et al., 2016; Ren et al., 2008). For example, several recent studies in degraded ecosystems under mine tailings (Gomez‐Aparicio, 2009; Navarro‐Cano et al., 2017) or burnt ecosystems (Paniw et al., 2017) have shown important effects of nurse plants in restoring the vegetation depending on the functional traits of nurse and beneficiary species. Although these studies empirically showed that nurse plants affect vegetation restoration by allowing the recruitment and survival of beneficiary species, some challenging issues such as how to change the effects of nurse species depending on stress level, different beneficiary relatedness (i.e., functional or phylogenetic relatedness) and spatial scale are still unclear (Craft, 2016; Jankju, 2013; Padilla & Pugnaire, 2006).

The majority of studies have shown important effects of nurse plants on structuring biodiversity facets, including taxonomic, functional, and phylogenetic diversity, through effects on beneficiary relatedness and the promoting of recruitment and survival (e.g., Butterfield & Briggs, 2011; Callaway, 2007; Cavieres et al., 2013; Le Bagousse‐Pinguet et al., 2019; Madrigal‐Gonzalez et al., 2020; Soliveres & Maestre, 2014; Valiente‐Banuet et al., 2007). However, biodiversity facets beneath nurse canopies may or not may exhibit consistent patterns depending on how functional traits conserve across phylogeny, as well as on levels of environmental harshness. For example, different responses of functional and phylogenetic diversity to nurse plant effects could be related to (1) functional traits that are not phylogenetically conserved (Valiente‐Banuet & Verdú, 2013; Vega‐Alvarez et al., 2019), and (2) functional traits that are labile because they cannot exhibit positive effects of nurse plants on beneficiary relatedness with an increase in environmental severity (Bashirzadeh et al., 2022). In this regard, some meta‐synthetic studies (Gomez‐Aparicio, 2009; He et al., 2013) show that growth form and some physiological traits such as leaf carbon and nitrogen content may clearly reflect plant strategies in response to major stress factors, including land use, land degradation, and changing soil nature in degraded ecosystems such as limestone mines (Wang et al., 2021). In limestone mines, there is extreme ecological condition in soil by a stronger proportion of calcium carbonate (CaCO3), soluble salts and by its neutrality or even its alkalinity (up to pH = 8.5) that influence plant growth and survival through inhibiting the activity of micro‐organisms, impeding the formation and mineralization of humus, reducing the availability of nitrogen, iron, and phosphorus (Kolodziejek & Patykowski, 2015; Nakata, 2015). However, mechanisms promoted by nurse species on plants are not clearly recognized about functional traits related to extreme ecological conditions in degraded ecosystems such as limestone mines. However, the mechanisms promoted by nurse plants in restoring biodiversity are not clearly recognized in relation to such functional traits and stress factors.

Plant–plant interactions specifically influence biodiversity facets and beneficiary relatedness depending on the biodiversity facets and life forms of nurse species in particular (Pashirzad et al., 2019; Soliveres et al., 2012). For example, some studies indicated that cushion plants have the most important effects on taxonomic and phylogenetic diversity in alpine environments (Butterfield et al., 2013; Cavieres et al., 2013), whereas nurse grasses can strongly affect functional diversity by providing suitable microhabitats for beneficiary species with distant relatedness (Gastauer et al., 2018; Navarro‐Cano et al., 2014). However, nurse plant effects on biodiversity facets may or not may be consistent across different spatial scales. This is particularly problematic, because most inference of nurse plant's effects is based on studies at the patch scale, rather than at the higher spatial scale (Brooker et al., 2008; Soliveres et al., 2012). Moreover, biodiversity patterns are highly scale‐dependent and different conclusions about the relative importance of biotic and abiotic factors can result from biodiversity analyses at different spatial scales (Cavender‐Bares et al., 2006; Swenson et al., 2006).

Because of these issues, taxonomic, functional, and phylogenetic approaches in restoration ecology should be applied across different spatial scales to enhance the entire restoration process (Hipp et al., 2015; Pitz et al., 2015). However, such a framework has been rarely applied when studying the restoration of degraded ecosystems (Ren et al., 2008; Singh et al., 2012). We might, therefore, better understand the effects of nurse plants on the restoration of ecosystems by considering the features of beneficiary species beneath nurse plants with different life forms, their relationships at different ages of abandonment since the cessation of mining and at different spatial scales (Gastauer et al., 2013; Perring et al., 2015). The availability of restored sites at different ages of abandonment and different spatial scales will enable a field observation of chronosequence changes in vegetation regarding effects of plant–plant interactions for land restoration (Cadotte, 2013; Clark et al., 2012; Navarro‐Cano et al., 2014; Prach & Tolvanen., 2016).

Here, we analyzed the effects of plant–plant interactions on taxonomic, functional, and phylogenetic diversity at the plot and vegetation‐patch scales with respect to different ages of abandonment in a limestone mine located in northeastern Iran. Previous studies only addressed either the effects of abandonment age or the presence of nurse plants on biodiversity, especially taxonomic diversity (Soliveres & Maestre, 2014). We obtained information on the life forms of nurse plants (we found two dominant nurse plants with grass and shrub life forms) and functional trait values and phylogenetic relationships of the plant species and addressed the following questions: (i) Do all biodiversity facets exhibit significant variations with respect to the presence of nurse plants and different ages of land abandonment at plot scale? (ii) Are the effects of dominant nurse plants on biodiversity consistent across biodiversity facets, ages of land abandonment, and spatial scales considered? (iii) Does each nurse plant species have a specific effect on restoring plant biodiversity regarding different abandonment ages and spatial scales?

## 2 MATERIAL AND METHODS

### 2.1 Study area

Our study region consists of abandoned mine lands and nearby natural rangelands in Cement‐Shargh, northeastern Iran (centered around 59.7467 N, 36.4746 E). Elevation ranges from 1000 to 1180 m, generally increasing from south to north (Table 1; Figure S1). Mean annual precipitation ranges from 150 to 200 mm with a semi‐arid climate. The rain season is from Autumn (late October) to early Spring, with the main rain season occurring from February through April, with an average temperature of 13° C. Plant communities are typically dominated by herbaceous plants and shrubs.

**TABLE 1:** Three sites were used in this study with respect to different ages of abandonment since mining activities (recent abandonment, old abandonment, and control sites with relatively no mining activities) in Cement‐Shargh in northeastern Iran. All the sites were assessed in two different scales of vegetation (i.e., plot scale including 1 × 1 m^2^ plots with and without the presence of nurse species and patch scale considering vegetation beneath *Artemisia sieberi* and *Stipa arabica* and their open areas with 0.5 × 0.5 m^2^ plots)

Mining time	Coordinates N/E	Elevation	Number of analyzed plots	Sampling unit area
All	59.7467 N/ 36.4746 E	1000–1130	120/240	1 x 1 /0.5 x 0.5
old	59.7378 N/ 36.4792 E	1009	40	1 x 1
Recent	59.7383 N/ 36.4780 E	1025	40	1 x 1
Control	59.7380 N/ 36.4777 E	1045	40	1 x 1
Old	59.7378 N/ 36.4792 E	1009	80	0.5 x 0.5
Recent	59.7383 N/ 36.4780 E	1025	80	0.5 x 0.5
Control	59.7380 N/ 36.4777 E	1045	80	0.5 x 0.5

The dominant plant species are typically *Artemisia scoparia* Waldst. & Kitam., *Artemisia sieberi* Besser (Asteraceae), *Stipa arabica* Trin. & Rupr. (Poaceae), *Carex stenophylla* Wahlenb., (Cyperaceae), *Poa bulbosa* L. (Poaceae), *Taeniatherum caput*‐*medusae* (L.) Nevski (Poaceae), *Alhagi sp*. (Fabaceae), and *Astragalus verus* Olivier. (Fabaceae). Our analyses focused on the two most dominant plant species as nurse plants, *A*. *sieberi* Besser and *S*. *arabica* Trin. & Rupr., both of which occurred at all of the sites sampled. *Artemisia sieberi* is an allelopathic dwarf shrub with less tight and wide crown. Facilitative effects of *Artemisia* species on understory plant species were clearly observed especially under moderate environmental conditions (Jankju, 2013; Jankju & Ejtehadi, 2016). *Artemisia* species generally create suitable microenvironments for understory species by increasing soil moisture and decreasing the irradiation and air temperature compared with open areas (Jankju et al., 2008). However, allelopathic effects have been observed on seed germination and seedling establishment, and this may influence total species diversity (Jankju, 2013). *S*. *arabica* is a herbaceous perennial bunchgrass that can grow to 0.39 m and has facilitative effects on the growth, recruitment, and survival of other species (Jankju, 2013; Jankju et al., 2008; Soliveres et al., 2014).

### 2.2 Data collection

#### 2.2.1 Site selection

We selected three sites based on different ages of abandonment since mining. They included control sites with relatively no mining activities and natural vegetation, old abandoned sites with mining activities terminated 50 or more years ago, and recently abandoned sites with mining having occurred within the last 10 years (Table 1; see details in Appendix S1). Importantly, we selected sites with the least impact from cement pollution to reduce bias in our results. To examine abandonment age, we extracted the time of last mining activity for each site from aerial photographs and from information on mining time recorded by staffs at the Cement‐Shargh site.

#### 2.2.2 Vegetation sampling based on nurse plant effects and land abandonment ages

To evaluate nurse plant effects with respect to land abandonment ages (i.e., across three sites), we considered two spatial scales: (1) the plot scale and (2) the vegetation‐patch scale (Pashirzad et al., 2019; Soliveres et al., 2012, 2014) (Table1; see details in Appendix S1).

At the plot scale, 40 1 m^2^ plots were established within each study site, with respect to the presence/absence of dominant nurse plant species (i.e., *A*. *sieberi* and *S*. *arabica*) (we selected plots based on the presence/absence of dominant nurse species). In this regard, we developed 20 1 m^2^ plots including the nurse plant species, and 20 1 m^2^ plots were surveyed without nurse plant species to balance our sampling effort at each study site. At each study site, the number of plots established was determined based on sampling area and the species‐area curve, and approximate distances between plots were 50–100 m. Finally, we surveyed vegetation composition within each 1 m^2^ plot by recording the number of individuals of all present plants at the plot (Palmer et al., 2010). We identified 37 plant taxa in total in the studied sites (see details in Appendix S2). Vegetation data were collected in late spring (middle June) and early summer (early July) 2021.

We considered the area beneath the canopies *of A*. *sieberi* and *S*. *Arabica* as another spatial scale named the “vegetation‐patch scale” to evaluate nurse effects (i.e., microclimatic amelioration provided by nurse plants) on restoring the vegetation. To measure the nurse effects at the vegetation‐patch scale, we randomly selected two individuals of each nurse plant (i.e., *A*. *sieberi* and *S*. *Arabica*) in each 1m^2^ plot including nurse plant species, and the area beneath their canopies was sampled using 0.5 × 0.5 m quadrats. Then, the same number of quadrats were sampled in open areas adjacent to these nurse plants (hereafter *Stipa* patch, *Artemisia* patch, and Open patch) (see details in Appendix S1). To properly assess the nurse plant effects across different spatial scales, it is important that the vegetation‐patch scale is located within larger spatial scale (Pashirzad et al., 2019; Soliveres et al., 2012). The number of samples and patch cover (i.e., 0.5 × 0.5 m) at the vegetation‐patch scale were obtained through assessing species‐area curves and previous groundbreaking references that advise a range of 20 to 30 samples with 0.5 × 0.5 m cover beneath the canopies of nurse species as appropriate replication and sample size for vegetation sampling beneath grasses and dwarf shrubs (Soliveres et al., 2012, 2014). In total, 80 quadrats [i.e., 40 (20 *Stipa* patch +20 its open area) +40 (20 *Artemisia* patch +20 its open area)] were established in each study site (see details in Appendix S1). The abundance based on both measurements (i.e., number of individuals and species cover for all plant species) was recorded within each quadrat. However, we considered the number of individuals for plant species present in paired quadrats for further analyses, because species co‐occurrence generally favors the establishment of one or more plant individuals (Eibes et al., 2021), and outcomes of plant–plant interactions are significantly sensitive to this variable (Soliveres et al., 2014).

#### 2.2.3 Functional trait information

We assessed eight functional traits for plant species in our sites including leaf dry matter content (LDMC) (g), specific leaf area (SLA) (m^2^ g^−1^), leaf nitrogen (mg g^−1^), leaf carbon content (mg g^−1^), plant height (cm), seed mass (mg), growth form, and life span to measure functional diversity (not trait analyses) in both plot and vegetation‐patch scales. These functional traits represent resource acquisition, resource limitation, and reproductive investment (Drenovsky et al., 2010; Douma et al., 2012; Ostertag et al., 2014; Sonnier et al., 2012). In addition, these traits were introduced as key functional traits for plant species in degraded environments and are dependent on biotic interactions, environmental conditions, and human‐based disturbances (Lavorel, 2013; Lienin & Kleyer, 2012; Meng et al., 2015; Soliveres et al., 2014). Leaf dry matter content (LDMC), specific leaf area (SLA), leaf nitrogen, and carbon content are related to growth rate and litter quality (Cortez et al., 2007; Dechaine et al., 2014; Kazakou et al., 2006). Plant height, growth form, and seed mass are strongly related to the ability of plants to compete, demographic features such as longevity and survival, respectively (Cornelissen et al., 2003; Moles et al., 2009; Ostertag et al., 2014). Life span represents plant strategies and abilities including competitive or facilitative abilities (Rahmanian et al., 2019).

We obtained information on these plant functional traits from publicly available trait datasets (BIEN package in R (Maitner et al., 2018), TRY (Kattge et al., 2011), LEDA (Kleyer et al., 2008) and TR8 (Bocci, 2014). Functional trait information was more obtained from BIEN package than other publicly available trait databases (because trait information for plant species in majority of worldwide is more available in BIEN than other databases such as TRY and LEDA). In addition, we considered plant height based on the vegetative part, because reproductive parts in many plant species especially in grasses create some biased measurements based on their variations in different growth stages (Cavieres et al., 2013). When multiple measurements per species were available in these databases, we averaged trait values to create a species mean trait value. For some plant species (five plant species; see details in Appendix S2 in Supporting Information), we used genus‐level means when either species‐level data were not available or plants were only identified to genus level (Lammana et al., 2014). Plant species present within our study sites belonged to four growth forms (i) grass, (ii) grasslike including sedges and rushes (i.e., plant species classified into Cyperaceae and Juncaceae families) (Blair et al., 2014), (iii) shrub, and iv) herb (see details in Appendix S2).

#### 2.2.4 Phylogenetic information

We obtained a phylogeny of 37 plant species present in all studied sites based on the most up‐to‐date megaphylogeny for seed plants (Smith et al., 2018). We standardized the species names in our dataset according to The Plant List using the R package “Taxonstand” (Cayuela et al., 2012). Then, we used the R function V. PhyloMaker (Jin & Qian, 2019) to link the species names in our dataset with those in the megaphylogeny, and the scenario 3 approach (Qian et al., 2016), to add species to the phylogeny. This phylogenetic tree was then used as reference lists from which phylogenetic diversity could be calculated for our communities and pairwise microsites in the dataset.

#### 2.2.5 Measures of species composition and plant functional groups

To measure the species composition, we performed detrended correspondence analysis (DCA) to illustrate the variation in plant species composition in different studied sites based on abundance data (i.e., plant individuals) (Legendre & Legendre, 2012). To illustrate species composition relationships with sites, ordination methods are more appropriate than clustering when the sites come from different land use forms (Cao et al., 2019; Taft & Kron, 2014). Then, we grouped plant species according to functional traits studied and illustrated major functional traits on two first components of DCA analysis to represent changes in plant species based on their functional groups across studied sites (Cao et al., 2019).

#### 2.2.6 Measures of taxonomic, functional, and phylogenetic diversity

To measure the effects of nurse plants on biodiversity facets across different spatial scales with respect to abandonment ages, we assessed biodiversity in both the plot (i.e., 1 m^2^ plots with and without considering nurse species) and vegetation‐patch scales (i.e., nurse species with grass and shrub life forms and their open areas). To measure taxonomic diversity, we used the first two Hill numbers to estimate species richness (q = 0) and species diversity as the exponential of Shannon's entropy (q = 1; referring to Shannon diversity) (Chao et al., 2014) for (i) the 1 m^2^ plots with and without nurse species and (ii) beneath *Stipa* and *Artemisia* patches and their open areas. The calculation was based on number of species, which is less affected by differences in total sampling effort than other methods (Chao & Jost, 2012).

To measure functional and phylogenetic diversity, we assessed abundance‐weighted mean pairwise distance (MPD) (Tucker et al., 2017; Webb et al., 2002) as the most robust measure of the phylogenetic and functional relatedness^76^ for (i) 1 m^2^ plots and (ii) paired patches (i.e., *Artemisia* and *Stipa* patches and their open patches). This index in the abundance‐weighted case is equivalent to Rao's Q and Hill numbers (Webb et al., 2002). Before measuring the functional diversity, we used Pearson correlation analysis to analyze the correlation between the traits. We did not find significant correlations between functional traits studied (see details in Appendix S3). Therefore, we considered all these functional traits in our further analyses and computed functional diversity by measuring the MPD index after the standardization of functional traits. Then, we calculated the standardized effect sizes of abundance‐weighted MPD for functional (FSES.mpd) and phylogenetic (PSES.mpd) diversity to produce a phylogenetic and functional index of diversity that is independent of species richness. We used the independent‐swap algorithm to draw a null distribution based on 999 randomizations and created standard effect sizes of mpd by comparing the observed community diversity to the null distribution of randomly assembled communities. Positive (SES values >1.96) and negative (SES values <−1.96) values of SES.mpd indicate significant functional/phylogenetic overdispersion and clustering patterns in the community, respectively. Functional/phylogenetic overdispersion structures represent important impacts of biotic interactions, such as competition between closely related taxa under benign conditions and facilitation between distantly related taxa under moderate conditions in plant communities. Functional/phylogenetic clustering represents important effects of abiotic factors in structuring the plant communities under extreme conditions (Soliveres et al., 2012, 2015). We calculated SES.mpd with the “ses.mpd” functions in R package picante (Kembel et al., 2010).

### 2.3 Statistical analyses

We analyzed variation in all biodiversity facets relative to i) the presence of nurse plants and abandonment age at plot scale and ii) different patches (i.e., *Stipa* patch and *Artemisia* patch) and abandonment age at vegetation‐patch scale. Differences in biodiversity indices were calculated across all studied sites. To measure differences in plant biodiversity, we used Kruskal–Wallis tests followed by Dunn's post hoc tests with Bonferroni correction (Haselberger et al., 2021) by subtracting control sites from sites in different ages of abandonment and comparing across all studied sites with available data (Haselberger et al., 2021).

The Wilcoxon–Mann–Whitney test (Wilcoxon, 1945) was performed with the R package ggpubr (Kassambara & Kassambara, 2020) to highlight differences in plant biodiversity (i) between plots with respect to the presence of nurse plants in each study site at plot scale and (ii) between paired patches (i.e., nurse patches and their open patches) in each study site at vegetation‐patch scale.

To assess the impacts of nurse plants, land abandonment age, and interactions on biodiversity indices at the plot and vegetation‐patch scales, we performed variation partitioning based on partial linear regression using the “varpart” function (Oksanen et al., 2016). The total percentage of variation explained was divided into unique and shared contributions for two predictors: (1) nurse plants (i.e., presence of nurse plants at plot scale and *Artemisia* patch and *Stipa* patch at vegetation‐patch scale) (pink fraction), (2) land abandonment age (i.e., Aband. Age factor) (blue fraction), and (3) shared contributions of both factors (shared area between blue and pink fractions). Analyses were conducted in R ver. 4.0.0, and figures were produced using the ggplot2 package (Wickham, 2011).

## 3 RESULTS

### 3.1 Species composition and plant functional groups under sites with different ages of land abandonment

**FIGURE 1:**
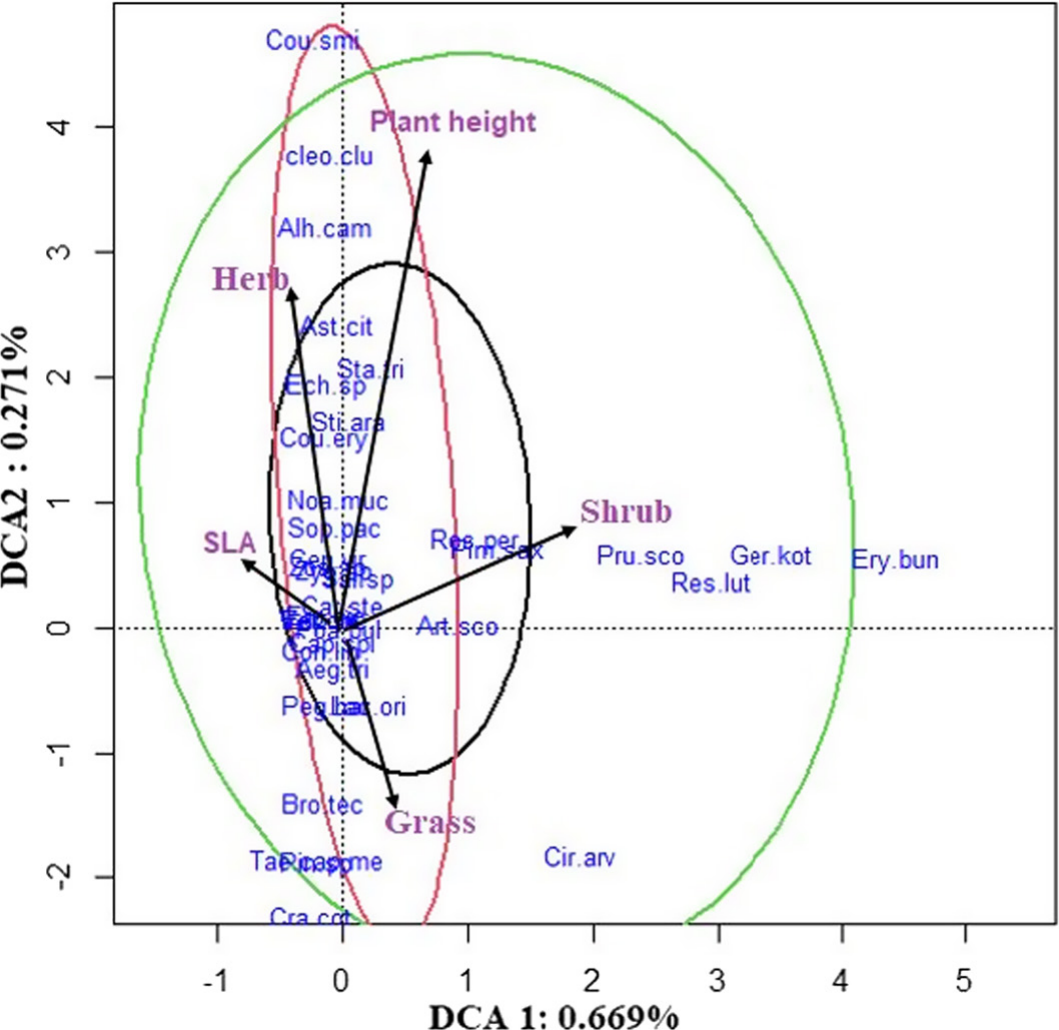
DCA ordination of plant composition and plant functional groups among three studied sites; control site (red circle), recent abandonment site (black circle), and old abandonment site (green circle). Qualitative traits were transformed into dummy variables or fuzzy coded variables (see Text). Abbreviations represent plant species names (species names in full details are included in Table S1). Vectors illustrate the correlation of plant functional groups with the first and second axes

Species composition and plant functional groups differed significantly between sites with different land abandonment ages (Figure 1). In old abandoned sites, we found a wide range of plant species (35 plant species) with different functional identities, such as *Prunus scoparia* (Spach) C.K. Schneid., *Artemisia sieberi* Besser (shrub group), *Bromus tectorum* L., *Poa bolbusa* L., *Taeniatherum caput*‐*medusae* (L.) Nevski (grass group), *Artemisia scoparia* Waldst. & Kitam., and *Cousinia eryngioides* Boiss. (herb group) (Figure 1; old aband. site; for more details, see Appendix S2 in Supporting Information). In contrast, herbaceous and grass plants (22 plant species), including *Peganum harmala* L., *Sophora pachycarpa* C.A. Mey., *Stipa arabica* Trin. & Rupr., *Carex stenophylla* Wahlenb., and *Rosa persica* L., were dominantly observed in recently abandoned sites (Figure 1; Recent aband. site). Although we found a higher number of plant species (26 plant species) in control sites than recent abandonment site, those were more located in herb and grass functional groups with similar SLA and plant height, including *Cousinia smirnowii* Trautv., *Astragalus crinitus* Boiss., *Centaurea virgata* Lam. and *Noaea mucronata* (Forssk.) Asch. & Schweinf., *Taeniatherum caput*‐*medusae* (L.) Nevski and *Poa bolbusa* L., rather than the shrub group, including *Artemisia sieberi* Besser and *Rosa persica* L. (Figure 1; Control site; for more details, see Appendix S2).

### 3.2 Effects of plant–plant interactions and abandonment ages on biodiversity facets at the plot scale

**FIGURE 2:**
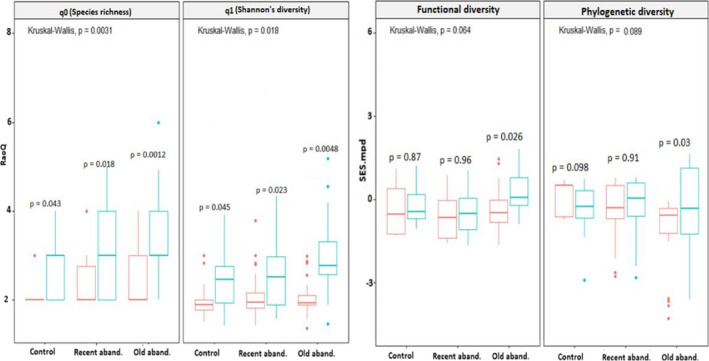
Boxplots showing differences in taxonomic (i.e., species richness (q0) and Shannon's diversity) (q1), functional (FSES.mpd) and phylogenetic (PSES.mpd) diversity at plot scale among three studied sites with respect to the presence (blue box; *n* = 20 1 m^2^ plots in each study site) or absence (red box; *n* = 20 1 m^2^ plots in each study site) of nurse species and different ages of abandonment. Box edges indicate the 25th–75th percentile for each variable. Error bars indicate 5th and 95th percentiles. Significant differences across all studied sites and between studied sites with respect to abandonment age after mining activities are reported from Kruskal–Wallis tests followed by Dunn's post hoc tests with Bonferroni correction. In addition, significant differences between plots with respect to the presence of nurse species are reported from the Wilcoxon–Mann–Whitney test as *p < .05, **p < .01, and ***p < .001

Taxonomic diversity indices at the plot scale responded significantly to the presence of nurse plants and land abandonment age (Figure 2; q0 = Kruskal–Wallis H *p* < .0031, q1 = *p* < .018), while functional and phylogenetic diversity marginally responded to the study factors (Figure 2; FSES.mpd = Kruskal–Wallis H *p* < .064, PSES.mpd = *p* < .089). A common result across all study sites was that higher taxonomic was observed in plots with nurse plants than plots without them, with significant variation between plots across all study sites (Figure 2; q0 and q1 panels). However, these differences were stronger in sites with old abandonment ages for all biodiversity indices (Figure 2). For functional and phylogenetic indices, we found an increase in functional and phylogenetic diversity (i.e., functional and phylogenetic overdispersion) in plots with nurse species, with significant and more consistent variations between the plots (i.e., plots with the presence and absence of nurse species) only in sites with old abandonment ages (Figure 2; FSES.mpd and PSES.mpd).

### 3.3 Effects of plant–plant interactions and abandonment ages on biodiversity facets at the vegetation‐patch scale

**FIGURE 3:**
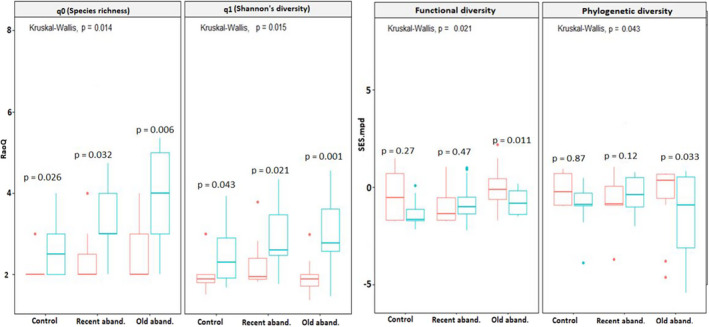
Boxplots showing differences in taxonomic (i.e., species richness (q0) and Shannon's diversity) (q1), functional (FSES.mpd) and phylogenetic (PSES.mpd) diversity at vegetation‐patch scale among three studied sites with different ages of abandonment and among different patches [i.e., Artemisia patches (blue box; *n* = 20 0.5 × 0.5 m quadrats in each study site) and their open patches (red box; *n* = 20 0.5 × 0.5 m quadrats in each study site)]. Box edges indicate the 25th–75th percentile for each variable. Error bars indicate the 5th and 95th percentiles. Significant differences across all studied sites with respect to abandonment age and the presence/absence of nurse shrubs are reported from Kruskal–Wallis tests followed by Dunn's post hoc tests with Bonferroni correction. In addition, significant differences between Artemisia patches and their open patches are reported from the Wilcoxon–Mann–Whitney test as *p < .05, **p < .01, and ***p < .001

**FIGURE 4:**
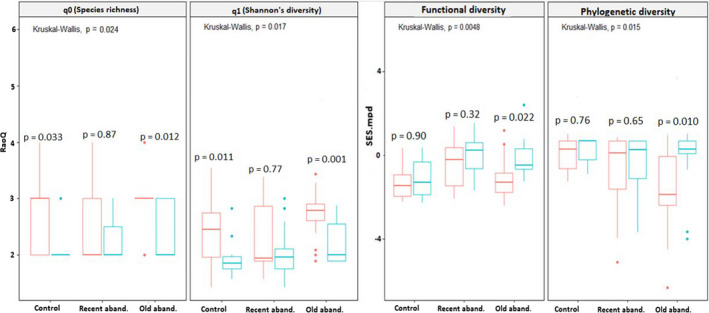
Boxplots showing differences in taxonomic (i.e., species richness (q0) and Shannon's diversity) (q1), functional (FSES.mpd) and phylogenetic (PSES.mpd) diversity at vegetation‐patch scale among three studied sites with different ages of abandonment and among different patches [i.e., Stipa patches (blue box; *n* = 20 0.5 × 0.5 m quadrats in each study site) and their open patches (red box; *n* = 20 0.5 × 0.5 m quadrats in each study site)]. Box edges indicate the 25th–75th percentile for each variable. Error bars indicate the 5th and 95th percentiles. Significant differences across all studied sites with respect to abandonment age and the presence/absence of nurse grasses are reported from Kruskal–Wallis tests followed by Dunn's post hoc tests with Bonferroni correction. In addition, significant differences between Stipa patches and their open patches are reported from the Wilcoxon–Mann–Whitney test as *p < .05, **p < .01, and ***p < .001

At the vegetation‐patch scale, all biodiversity facets differed with abandonment age and nurse plant life form. Generally, taxonomic, functional, and phylogenetic diversity in sites with *Stipa* patches differed from sites with *Artemisia* patches (Figures 3, 4). In this regard, the impacts of nurse plants across land abandonment ages were stronger for taxonomic diversity in *Artemisia* patches (Figure 3; q0 = Kruskal–Wallis H *p* < .0031, q1 = *p* < .018), and for functional and phylogenetic diversity in *Stipa* patches (Figure 4; FSES.mpd = Kruskal–Wallis H *p* < .0031, PSES.mpd = *p* < .018).

We found significantly higher taxonomic diversity in *Artemisia* patches than in nearby open patches (Figure 3; q0 = *p* < .014, q1 = *p* < .015), especially in old abandoned sites. In contrast, we found greater impacts of *Artemisia* patches on functional and phylogenetic diversity in old abandoned sites (Figure 3; old abandoned sites, *p* < .01), with more likely significant functional/ phylogenetic clustering under *Artemisia* patches than nearby open patches (Figure 3; FSES.mpd, PSES.mpd).

*Stipa* patches had strong, negative effects on taxonomic diversity in all study sites (Figure 4, q0 = *p* < .024 and q1 = *p* < .017), with more monotonic and negative impacts in old abandoned sites than other sites studied (Figure 4, old abandoned sites, *p* < .01). We found more significant differences in taxonomic diversity between *Stipa* patches and their open patches in old abandoned sites in comparison with other studied sites (Figure 4, q0 = *p* < .04, q1 = *p* < .011). In contrast, functional and phylogenetic diversity in *Stipa* patches was significantly enhanced in all studied sites (Figure 4; FSES.mpd and PSES.mpd). However, more significant differences between *Stipa* patches and nearby open patches for functional and phylogenetic diversity were observed in old abandoned sites, with increasing functional and phylogenetic overdispersion patterns in *Stipa* patches compared with open patches (Figure 4; FSES.mpd and PSES.mpd).

### 3.4 The contributions of study factors on biodiversity variation at different spatial scales

**FIGURE 5:**
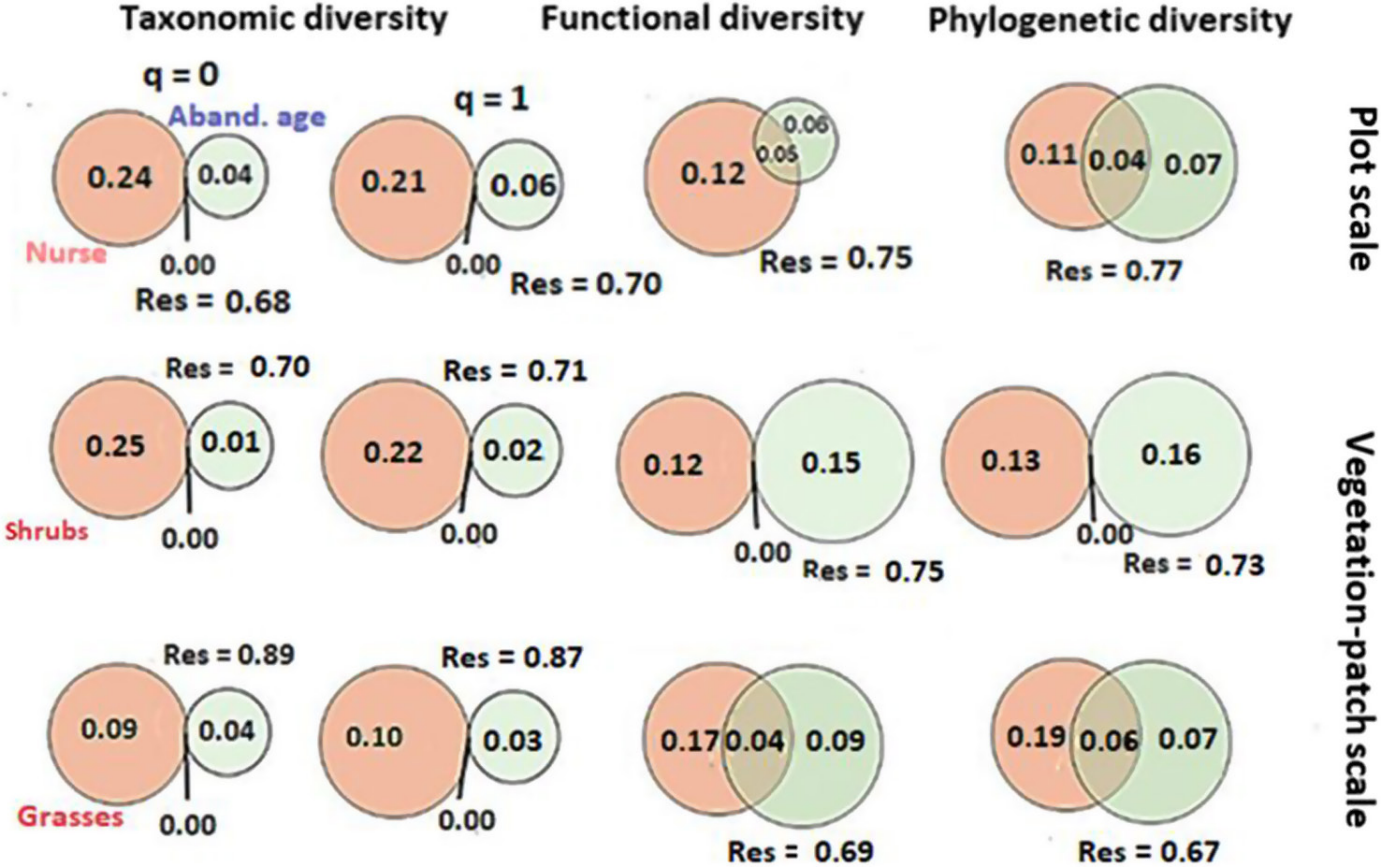
Relative contribution of land abandonment ages (in blue; i.e., Aband. age factor) and (i) the presence of nurse plants at plot scale (in pink; i.e., Nurse) and (ii) the presence of nurse plants with different life forms (in pink; i.e., grasses and shrubs) at vegetation‐patch scale to taxonomic (q0 and q1 indices), functional (FSES.mpd) and phylogenetic (PSES.mpd) diversity. Values represent the adjusted R^2^‐values

The amount of variance explained for all diversity facets increased when considering nurse species at both spatial scales (pink fraction in Figure 5). This was particularly true for all biodiversity indices at plot scale and for taxonomic diversity and functional and phylogenetic diversity with respect to *Artemisia* patches and *Stipa* patches, respectively, at vegetation‐patch scale (please see pink fraction in Figure 5). In addition, land abandonment age was a particularly strong predictor of the variation in phylogenetic and functional diversity in *Artemisia* patches (Figure 5; blue fraction). Interactions between the presence of nurse plants and land abandonment age (see shared area between pink and blue fractions) were also the most important predictors of variation in phylogenetic and functional diversity at the plot scale and of functional and phylogenetic diversity in *Stipa* patches (Figure 5; see shared area between pink and blue fractions in plot and *Stipa* patch scales), suggesting that the effects of plant–plant interactions on these biodiversity facets are relatively sensitive to land abandonment ages.

## 4 DISCUSSION

Biodiversity facets at the plot scale differed significantly with the presence of nurse plants across ages of land abandonment. In this regard, a significant increase in taxonomic and functional/phylogenetic diversity was observed, when considering plots with nurse species than plots without nurse species. Such differences in taxonomic and functional/phylogenetic diversity were consistent with biodiversity patterns beneath Artemisia and Stipa patches. In this regard, dominant shrub (i.e., *A*. *sieberi*) strongly enhanced the presence of plant species (i.e., an increase in species richness (q0)), whereas dominant grass (i.e., *S*. *arabica*) promoted some specific mechanisms to enhance functional and phylogenetic relatedness between beneficiary species. These results suggest the importance of nurse‐based approaches in restoring vegetation at different spatial scales of degraded ecosystems (Landero & Valiente‐Banuet, 2010; Navarro‐Cano et al., 2017; Ren et al., 2008). Nurse‐based approaches explain that increase in biodiversity facets is due to the presence of suitable microhabitats beneath the nurse plants, depending on their morphological features and their positive or negative effects on beneficiary species (Gomez‐Aparicio, 2009; Soliveres et al., 2012, 2015). However, our results also suggest that different nurse species can restore plant biodiversity after degradation only in places that are currently under long ages of land abandonment (Navarro‐Cano et al., 2017; Soliveres et al., 2014). It might be associated with a significant decrease in harmful variations in soil nature such as a decrease in proportion of calcium carbonate (CaCO3), soluble salts, and its alkalinity (up to pH = 8.5) under long ages of land abandonment after mining activities (Kolodziejek & Patykowski, 2015; Nakata, 2015). Plant growth and survival are significantly affected by CaCO3 and basic pH, and a decrease in these factors by effects of nurse plants under long ages of land abandonment can lead to an increase in taxonomic diversity and species richness (Navarro‐Cano et al., 2017). Therefore, our results represent the effects of nurse plants on restoring vegetation depending on land abandonment ages and biodiversity facet considered.

### 4.1 Plant biodiversity through the presence of nurse plants and land abandonment ages at multiple spatial scales

We found stronger effects of the presence of nurse plants than land abandonment ages on biodiversity facets at the plot scale. This result was consistent with the significant effects of *Artemisia* patches on taxonomic diversity and *Stipa* patches on functional and phylogenetic diversity at the vegetation‐patch scale. Therefore, this finding shows that nurse plants can enhance plant biodiversity at the plot scale by providing suitable microhabitats for beneficiary species and promoting specific mechanisms depending on life form and biodiversity facet (Gomez‐Aparicio, 2009). It seems that an increased taxonomic diversity in *Artemisia* patches and functional and phylogenetic diversity in *Stipa* patches are influenced by the presence of a wide range of plant species or by some specific plant species with different functional identities and distant evolutionary relatedness. However, some recent studies have shown negative or neutral effects of *Artemisia* patches on plant biodiversity structure, especially on taxonomic diversity (Bahalkeh et al., 2021; Rahmanian et al., 2021). These contrasting results are likely to be contingent on different environmental conditions. In our study area, there are numerous severe environmental conditions caused by mining activities, including harmful changes in soil nature and properties and some extreme climatic conditions. It is possible that nurse types in such extreme environments can better play their facilitative roles on different facets of biodiversity than natural environments (Gomez‐Aparicio, 2009; Navaro‐Cano et al., 2017).

An increase in biodiversity facets consistent with the significant positive effects of *Artemisia* and *Stipa* patches on them was only observed under moderate disturbance conditions (i.e., old abandoned site). In contrast, the negative impacts of nurse plants on plant biodiversity were observed under severe environmental conditions (i.e., recent abandoned site). Therefore, we strongly confirm that positive impacts of nurse species are significantly associated with environmental or disturbance level considered (Bashirzadeh et al., 2022; Soliveres et al., 2015). In this regard, our results provide strong empirical support for positive impacts of nurse species to present a wide range of plant species under moderate disturbance conditions in concordance with stress‐gradient hypothesis (Michalet et al., 2006), and secondary succession as an important restorative process (Maestre & Cortina, 2004).

### 4.2 Effects of nurse types on restoration of biodiversity facets

The effects of different patches on specific biodiversity facets suggest that there are likely impacts of the morphological features of nurse plants, as well as some processes controlling beneficiary diversity, on the restoration of plant biodiversity (Soliveres et al., 2012; Valiente‐Banuet & Verdu, 2013). A significant increase in taxonomic diversity beneath *Artemisia* patches (see q0 and q1 in Figure 2) under moderate disturbance condition could be a consequence of specific shrub canopy characteristics on beneficiary species. *A*. *sieberi* shrubs have large canopy areas, which may provide a large suitable microhabitat to facilitate a large number of beneficiary species (Jankju et al., 2013). Additionally, the porous canopy of *A*. *sieberi* may promote greater spatial segregation between beneficiary species (Pistón et al., 2016), reducing competition between them. Such spatial segregation between understory species may allow greater coexistence without the need for strong trait or evolutionary differences (Losapio et al., 2021). Our results provided strong support for these explanations, illustrating an increase in taxonomic diversity and a decrease in functional/phylogenetic relatedness between plant species in *Artemisia* patches. In addition, our findings and some previous studies (Jankju et al., 2008; Perring et al., 2015) confirm that allelopathic effects of *Artemisia* plant in one successional stage may facilitate the presence of plants in the next successional stage. In this regard, although there is an explanation about excluding some plant species via *Artemisia's* allelopathic effects (Jankju, 2013; Rahmanian et al., 2021), a vast suitable area beneath *Artemisia* due to niche partitioning will provide an increase in species richness (Bashirzadeh et al., 2022). However, niche partitioning in the *Artemisia* patches in our study was only observed under moderate disturbance conditions. In this regard, there is a strong support for stress‐gradient hypothesis that states some morphological and allelopathic features of *Artemisia* patches are significantly observed under moderate conditions (Aerts et al., 1991; Jankju, 2013; Köchy & Wilson, 2000; Luiz et al., 2013; Pistón et al., 2016).

An increase in functional/phylogenetic diversity under *Stipa* patches may be caused by negative interactions between beneficiary species (Goldberg et al., 2001; Winkler et al., 2014). Grasses generally provide a specific microhabitat based on their competitive nature, surface roots, dense and small canopy area (Craft, 2016; Padilla & Pugnaire, 2006). In such microhabitat, negative interactions such as competition will occur between beneficiary species. In addition, nurse grasses also compete efficiently with beneficiary species based on their morphological features, including fibrous roots and a large root: shoot ratio (Gomez‐Aparicio, 2009; Zhenqi et al., 2012). Such negative interactions may lead to niche divergence among beneficiary species by enhancing the degree of overdispersion among traits and so decreasing evolutionary relatedness (Butterfield & Briggs, 2011; Gastauer et al., 2018; Gavini et al., 2019). Thus, fewer, more functionally or phylogenetically divergent, species may be able to coexist through interactions between nurse grasses with beneficiary species (Navarro‐Cano et al., 2017) (Figure 3; a decrease in taxonomic diversity and an increase in functional/phylogenetic diversity are illustrated in *Stipa* patches). However, such competition between interacting species will significantly occur when disturbance conditions are moderate (i.e., under old abandoned site that plant species with different functional identities such as herbs and grasses with different SLA and plant height were observed) (Soliveres et al., 2012, 2014). This explanation is consistent with our variation partitioning results, which suggest important impacts of land abandonment age and the presence of nurse grasses on plant biodiversity (Butterfield & Briggs, 2011; Gavini et al., 2019).

### 4.3 Conservation and management strategies based on our new and novel findings

Our study provided some new and novel results regarding the important impacts of nurse species in restoring vegetation in a disturbed ecosystem. Our findings are: i) depending on morphological characteristics of nurse species and disturbance level, nurse plants can promote specific effects in restoring vegetation. For example, we found nurse shrubs enhanced the presence of plant species with different functional identities, while nurse grasses promoted some specific mechanisms such as an increase in functional and phylogenetic relatedness between beneficiary species based on competitive ability. In addition, all of these positive effects were observed under moderate disturbance conditions (i.e., under old abandoned site) and were consistent across larger spatial scales. In this regard, our findings show the benefits of assessing the functional and phylogenetic facets of biodiversity to recognize the nurse plant's effects and their mechanisms on restoring the vegetation. Functional and phylogenetic facets could promote better interpretations about plant species relatedness beneath nurse canopies. Therefore, we believe that our results have the potential to foster new research and more detailed and large analyses investigating nurse‐based approaches in vegetation restoration. In this case, we suggest developing some projects based on planting shrubs and grasses in disturbed ecosystems to promote vegetation restoration.

## 5 CONFLICT OF INTERESTS

The authors declare that they have no conflict of interests.

## AUTHOR CONTRIBUTION

**Maral Bashirzadeh:** Data curation (equal); Formal analysis (equal); Investigation (equal); Methodology (equal); Project administration (equal); Resources (equal); Software (equal); Validation (equal); Visualization (equal); Writing – original draft (equal); Writing – review & editing (equal). **Richard P Shefferson:** Conceptualization (equal); Methodology (equal); Validation (equal); Writing – review & editing (equal). **Mohammad Farzam:** Data curation (equal); Project administration (equal); Supervision (equal); Writing – review & editing (equal).

## AUTHOR CONTRIBUTION

M. F. designed the study; M.B. and M.F. provided the data; data analyses and preparation of figures and tables were done by M.B.; the paper was written by M.B. and R. P. S.; and all authors substantially contributed to the subsequent drafts.

## Supporting information

Appendix S1‐S4

## Data Availability

All data supporting this study are available at Appendix S4 in Supporting Information file.

## References

[ece38878-bib-0001] Adesipo, A. A. , Freese, D. , Zerbe, S. , & Wiegleb, G. (2021). An approach to thresholds for evaluating post‐mining site reclamation. Sustainability, 13, 5618. 10.3390/su13105618

[ece38878-bib-0113] Aerts, R. , Boot, R. G. A. , & Van Der Aart, P. J. M. (1991). The relation between above‐ and belowground biomass allocation patterns and competitive ability. Oecologia, 87, 551–559. 28313698 10.1007/BF00320419

[ece38878-bib-0002] Bahalkeh, K. , Abedi, M. , Dianati, G. A. , & Michalet, R. (2021). *Artemisia sieberi* shrubs have contrasting specific effects on understory species in Iranian steppes. Applied Vegetation Science, 32(4), e13067.

[ece38878-bib-0003] Bashirzadeh, M. , Soliveres, S. , Farzam, M. , & Ejtehadi, H. (2022). Plant–plant interactions determine taxonomic, functional and phylogenetic diversity in severe ecosystems. Global Ecology and Biogeography, 31(4), 649–662.

[ece38878-bib-0004] Blair, J. , Nippert, J. , & Briggs, J. (2014). Grassland Ecology. Springer, pp. 9–14, 10.1007/978-1-4614-7501

[ece38878-bib-0005] Bocci, G. (2008). TR8: an R package for easily retrieving plant species traits. Methods in Ecology and Evolution, 6(3), 347–450. 10.1111/2041-210X.12327

[ece38878-bib-0006] Bradshaw, A. (2000). The use of natural processes in reclamation advantages and difficulties. Landscape and Urban Planning, 51, 89–100. 10.1016/S0169-2046(00)00099-2

[ece38878-bib-0101] Brooker, R. W. , Maestre, F. T. , Callaway, R. M. , Lortie, C. L. , Cavieres, L. A. , Kunstler, G. , Liancourt, P. , Tielbörger, K. , Travis, J. M. , Anthelme, F. , & Armas, C. (2008). Facilitation in plant communities: The past, the present, and the future. Journal of Ecology, 96, 18–34.

[ece38878-bib-0007] Butterfield, B. J. , & Briggs, J. M. (2011). Regeneration niche differentiates functional strategies of desert woody plant species. Oecologia, 165(2), 477–487. 10.1007/s00442-010-1741-y 20686787 PMC3021705

[ece38878-bib-0100] Butterfield, B. J. , Cavieres, L. A. , Callaway, R. M. , Cook, B. J. , Kikvidze, Z. , Lortie, C. J. , Michalet, R. , Pugnaire, F. I. , Schöb, C. , Xiao, S. , Zaitchek, B. , Anthelme, F. , Björk, R. G. , Dickinson, K. , Gavilán, R. , Kanka, R. , Maalouf, J.‐P. , Noroozi, J. , Parajuli, R. , … Brooker, R. W. (2013). Alpine cushion plants inhibit the loss of phylogenetic diversity in severe environments. Ecology Letters, 16(4), 478–486. 23346919 10.1111/ele.12070

[ece38878-bib-0008] Cadotte, M. W. (2013). Experimental evidence that evolutionarily diverse assemblages result in higher productivity. Proceedings of the National Academy of Sciences, 110, 8996–9000. 10.1073/pnas.1301685110 PMC3670319 23674676

[ece38878-bib-0009] Callaway, R. M. (2007). Positive interactions and interdependence in plant communities. Springer.

[ece38878-bib-0011] Cao, G. , Tsuchiya, K. , Zhu, W. , & Okuro, T. (2019). Vegetation dynamics of abandoned paddy fields and surrounding wetlands in the lower Tumen River Basin, Northeast China. PeerJ, 7, e6704– 10.7717/peerj.6704 30993042 PMC6459177

[ece38878-bib-0012] Cavender‐Bares, J. , Kozak, K. H. , Fine, P. V. A. , & Kembel, S. W. (2006). The merging of community ecology and phylogenetic biology. Ecology Letters, 12, 693e715. 10.1111/j.1461-0248.2009.01314.x 19473217

[ece38878-bib-0013] Cavieres, L. A. , Brooker, R. W. , Butterfield, B. J. , Cook, B. J. , Kikvidze, Z. , Lortie, C. J. , Michalet, R. , Pugnaire, F. I. , Schöb, C. , Xiao, S. , & Anthelme, F. (2013). Facilitative plant interactions and climate simultaneously drive alpine plant diversity. Ecology Letters, 17(2), 193–202. 24238015 10.1111/ele.12217

[ece38878-bib-0014] Cayuela, L. , De la Cerda, I. G. , Albuquerque, F. S. , & Golicher, D. J. (2012). taxonstand: An r package for species names standardization in vegetation databases. Methods. Ecology and Evolution, 3(6), 1078–1083.

[ece38878-bib-0110] Chao, A. , Gotelli, N. J. , Hsieh, T. C. , Sander, E. L. , Ma, K. H. , Colwell, R. K. , & Ellison, A. M. (2014). Rarefaction and extrapolation with Hill numbers: A framework for sampling and estimation in species diversity studies. Ecological Monographs, 84, 45–67.

[ece38878-bib-0015] Chao, A. , & Jost, L. (2012). Coverage‐based rarefaction and extrapolation: Standardizing samples by completeness rather than size. Ecology, 93, 2533–2547. 10.1890/11-1952.1 23431585

[ece38878-bib-0016] Clark, C. M. , Flynn, D. F. B. , Butterfield, B. J. , & Reich, P. B. (2012). Testing the link between functional diversity and ecosystem functioning in a Minnesota grassland experiment. PLoS One, 7(12), e52821. 10.1371/journal.pone.0052821 23300787 PMC3534119

[ece38878-bib-0017] Conesa, H. M. , & Schulin, R. (2010). The Cartagena‐La Unión mining district (SE Spain): A review of environmental problems and emerging phytoremediation solutions after fifteen years research. Journal of Environmental Monitoring, 12, 1225–1233. 10.1039/c000346h 20390210

[ece38878-bib-0108] Cornelissen, J. H. , Lavorel, S. , Diaz, S. , & Garnier, E. B. (2003). Handbook of protocols for standardised and easy measurement of plant functional traits worldwide. Australian Journal of Botany, 51(4), 335–380.

[ece38878-bib-0107] Cortez, M. C. , Silva, F. , & Areal, N. (2009). Socially responsible investing in the global market: The performance of us and European funds. International Journal of Finance & Economics, 17(3), 1–37.

[ece38878-bib-0018] Craft, C. (2016). Creating and restoring wetlands, from theory to practice. Elsevier, 1–348.

[ece38878-bib-0019] Cuevas, J. G. , Silva, S. I. , León‐Lobos, P. , & Ginocchio, R. (2013). Nurse effect and herbivory exclusion facilitate plant colonization in abandoned mine tailings storage facilities in north‐central Chile. Revista Chilena De Historia Natural, 86, 63–74. 10.4067/S0716-078X2013000100006

[ece38878-bib-0020] Dechaine, E. G. , Wendling, B. M. , & Forester, B. R. (2014). Integrating environmental, molecular, and morphological data to unravel an ice‐age radiation of arctic‐alpine Campanula in western North America. Ecology and Evolution, 4, 3940–3959. 25505522 10.1002/ece3.1168 PMC4242577

[ece38878-bib-0021] Douma, J. C. , Bardin, V. , Bartholomeus, R. P. , & Bodegom, P. M. (2012). Quantifying the functional responses of vegetation to drought and oxygen stress in temperate ecosystems. Journal of Functional Ecology, 26(6), 1355–1365.

[ece38878-bib-0105] Drenovsky, R. E. , Grewell, B. J. , Dantanio, C. M. , & Funk, J. L. (2012). A functional trait perspective on plant invasion. Annals of Botany, 110(1), 141–153. 22589328 10.1093/aob/mcs100 PMC3380596

[ece38878-bib-0022] Eibes, P. M. , Eisenbacher, J. , Beierkuhnlein, C. , Chiarucci, A. , Field, R. , Jentsch, A. , Köhler, T. , Vetaas, O. R. , & Irl, S. D. H. (2021). Co‐occurrence frequency in vegetation patches decreases towards the harsh edge along an arid volcanic elevational gradient. Frontiers in Biogeography, 13(3), e49743. 10.21425/F5FBG49743

[ece38878-bib-0023] European Commission . (2016). Reference document on best available techniques for management of tailings and waste‐rock in mining activities. Retrieved from http://eippcb.jrc.ec.europa.eu/reference/BREF/mmr_adopted_0109.pdf (2016)

[ece38878-bib-0024] Gastauer, M. , Silva, J. R. , Caldeira Junior, C. F. , Ramos, S. J. , Souza Filho, P. W. M. , Furtini Neto, A. E. , & Siqueira, J. O. (2018). Mine land rehabilitation: Modern ecological approaches for more sustainable mining. Journal of Cleaner Production, 172, 1409–1422. 10.1016/j.jclepro.2017.10.223

[ece38878-bib-0025] Gastauer, M. , Trein, L. , Meira‐Neto, J. A. A. , & Schumacher, W. (2013). Evaluation of biotope's importance for biotic resource protection by the Bonner Approach. Ecological Indicators, 24, 193–200. 10.1016/j.ecolind.2012.06.014

[ece38878-bib-0026] Gavini, S. S. , Suárez, G. M. , Ezcurra, C. , & Aizen, M. A. (2019). Facilitation of vascular plants by cushion mosses in high‐Andean communities. Alpine Botany, 129(2), 137–148.

[ece38878-bib-0027] Giannini, T. C. , Giulietti, A. M. , Harley, R. M. , Viana, P. L. , Jaffe, R. , Alves, R. , Pinto, C. E. , Mota, N. F. , Caldeira Jr, C. F. , Furtini, A. E. , & Imperatriz‐Fonseca, V. L. (2016). Selecting plant species for practical restoration of degraded lands using a multiple‐trait approach. *Australian* . Ecology, 42, 510e521.

[ece38878-bib-0115] Goldberg, D. E. , Turkington, R. , Olsvig‐Whittaker, L. , & Dyer, A. R. (2001). Density dependence in an annual plant community: Variation among life history stages. Ecological Monographs, 71, 423–446.

[ece38878-bib-0028] Gomez‐Aparicio, L. (2009). The role of plant interactions in the restoration of degraded ecosystems: a meta‐analysis across life‐forms and ecosystems. Journal of Ecology, 97, 1202–1214. 10.1111/j.1365-2745.2009.01573.x

[ece38878-bib-0029] Haselberger, S. , Ohler, L. M. , Junker, R. R. , Otto, J. C. , Glade, T. , & Kraushaar, S. (2021). Quantification of biogeomorphic interactions between small‐scale sediment transport and primary vegetation. Earth Surface Processes and Landforms, 46, 1941–1952.

[ece38878-bib-0099] He, Q. , Bertness, M. D. , & Altieri, A. H. (2013). Global shifts towards positive species interactions with increasing environmental stress. Ecology Letters, 16, 695–706. 23363430 10.1111/ele.12080

[ece38878-bib-0030] Hipp, A. L. , Larkin, D. J. , Barak, R. S. , Bowles, M. L. , & Cadotte, M. W. (2015). Phylogeny in the service of ecological restoration. American Journal of Botany, 102, 647e648. 10.3732/ajb.1500119 26022478

[ece38878-bib-0032] Jankju, M. (2013). Role of nurse shrubs in restoration of an arid rangeland: effects of microclimate on grass Establishment. Journal of Arid Environment, 89, 103–109. 10.1016/j.jaridenv.2012.09.008

[ece38878-bib-0033] Jankju, M. , Delavari, A. , & Ganjali, A. (2008). Interseeding *Bromus kopetdaghensis*, in shrublands. Iranian Journal of Rangland Science, 2, 314–328.

[ece38878-bib-0034] Jankju, M. , & Ejtehadi, H. (2016). Effects of drought and slope aspect on canopy facilitation in a mountainous rangeland. Journal of Plant Ecology, 10(4), 626–633.

[ece38878-bib-0035] Jin, Y. , & Qian, H. (2019). V.PhyloMaker: an R package that can generate very large phylogenies for vascular plants. Ecography, 42, 1353–1359. 10.1111/ecog.04434 PMC9363651 35967255

[ece38878-bib-0036] Kassambara, A. , & Kassambara, M. A. (2020). Package ‘ggpubr’.

[ece38878-bib-0037] Kattge, J. , Díaz, S. , Lavorel, S. , Prentice, I. C. , Leadley, P. , Bönisch, G. , Garnier, E. , Westoby, M. , Reich, P. B. , Wright, I. J. , Cornelissen, J. H. C. , Violle, C. , Harrison, S. P. , Van BODEGOM, P. M. , Reichstein, M. , Enquist, B. J. , Soudzilovskaia, N. A. , Ackerly, D. D. , Anand, M. , … Wirth, C. (2011). TRY – a global database of plant traits. Global Change Biology, 17(9), 2905–2935. 10.1111/j.1365-2486.2011.02451.x

[ece38878-bib-0038] Kazakou, E. , Vile, D. , Shipley, B. , Gallet, C. , & Garnier, E. (2006). Co‐variations in litter decomposition, leaf traits and plant growth in species from a Mediterranean old‐field succession. Functional Ecology, 20(1), 21–30. 10.1111/j.1365-2435.2006.01080.x

[ece38878-bib-0039] Kembel, S. W. , Cowan, P. D. , Helmus, M. R. , Cornwell, W. K. , Morlon, H. , Ackerly, D. D. , Blomberg, S. P. , & Webb, C. O. (2010). Picante: R tools for integrating phylogenies and ecology. Bioinformatics, 26(11), 1463–1464. 10.1093/bioinformatics/btq166 20395285

[ece38878-bib-0040] Khater, C. , & Arnaud, M. (2007). Application of restoration ecology principles to the practice of limestone quarry rehabilitation in Lebanon. Lebanian Science Journal, 8(1), 19–28.

[ece38878-bib-0041] Kleyer, M. , Bekker, R. , Knevel, I. , Bakker, J. , Thompson, K. , Sonnenschein, M. , Poschlod, P. , Van Groenendael, J. , Klimes, L. , & Klimesova, J. (2008). The LEDA Traitbase: A database of life‐history traits of the Northwest European flora. Journal of Ecology, 96(6), 1266–1274.

[ece38878-bib-0042] Köchy, M. , & Wilson, S. D. (2000). Competitive effects of shrubs and grasses in prairie. Oikos, 91, 385–395. 10.1034/j.1600-0706.2000.910219.x

[ece38878-bib-0043] Kolodziejek, J. , & Patykowski, J. (2015). The effect of temperature, light and calcium carbonate on seed germination and radicle growth of the polycarpic perennial Galium cracoviense (Rubiaceae), a narrow endemic species from southern Poland. Acta Biologica Cracoviensia s. Botanica, 57(1), 70–81. 10.1515/abcsb-2015-0006

[ece38878-bib-0044] Lamanna, C. , Blonder, B. , Violle, C. , Kraft, N. J. , Sandel, B. , Šímová, I. , Donoghue, J. C. , Svenning, J.‐C. , McGill, B. J. , Boyle, B. , Buzzard, V. , Dolins, S. , Jørgensen, P. M. , Marcuse‐Kubitza, A. , Morueta‐Holme, N. , Peet, R. K. , Piel, W. H. , Regetz, J. , Schildhauer, M. , … Enquist, B. J. (2014). Functional trait space and the latitudinal diversity gradient. Proceedings of the National Academy of Sciences, 111, 13745–13750. 10.1073/pnas.1317722111 PMC4183280 25225365

[ece38878-bib-0045] Landero, J. P. , & Valiente‐Banuet, A. (2010). Species‐specificity of nurse plants for the establishment, survivorship, and growth of a columnar cactus1. American Journal of Botany, 97(8), 1289–1295. 21616881 10.3732/ajb.1000088

[ece38878-bib-0046] Lavorel, S. (2013). Plant functional effects on ecosystem services. Journal of Ecology, 101(1), 4–8.

[ece38878-bib-0047] Le Bagousse‐Pinguet, Y. , Soliveres, S. , Gross, N. , Torices, R. , Berdugo, M. , & Maestre, F. T. (2019). Phylogenetic, functional, and taxonomic richness have both positive and negative effects on ecosystem multifunctionality. Proceedings of National Academy Science USA, 116(17), 8419–8424. 10.1073/pnas.1815727116 PMC6486734 30948639

[ece38878-bib-0048] Legendre, L. , & Legendre, P. (2012). Numerical ecology. Elsevier.

[ece38878-bib-0049] Lienin, P. , & Kleyer, M. (2012). Plant trait responses to the environment and effects on ecosystem properties. Basic and Applied Ecology, 13(4), 301–311. 10.1016/j.baae.2012.05.002

[ece38878-bib-0051] Losapio, G. , Schöb, C. , Staniczenko, P. P. A. , Carrara, F. , Palamara, G. M. , De Moraes, C. M. , Mescher, M. C. , Brooker, R. W. , Butterfield, B. J. , Callaway, R. M. , Cavieres, L. A. , Kikvidze, Z. , Lortie, C. J. , Michalet, R. , Pugnaire, F. I. , & Bascompte, J. (2021). Network motifs involving both competition and facilitation predict biodiversity in alpine plant communities. Proceedings of National Academy Science USA, 118(6), e2005759118. 10.1073/pnas.2005759118 PMC8017722 33526655

[ece38878-bib-0114] Luiz, A. M. , Leao‐Pires, T. A. , & Sawaya, R. J. (2016). Geomorphology drives amphibian beta diversity in Atlantic forest Lowlands of southeastern Brazil. PLoS One, 11(5), e0153977. 27171522 10.1371/journal.pone.0153977 PMC4865194

[ece38878-bib-0052] Madrigal‐Gonzalez, J. , Cano‐Barbacil, C. , Kigel, J. , Ferrandis, P. , & Luzuriaga, A. L. (2020). Nurse plants promote taxonomic and functional diversity in an arid Mediterranean annual plant community. Journal of Vegetation Science, 31(4), 658–666. 10.1111/jvs.12876

[ece38878-bib-0053] Maestre, F. T. , & Cortina, J. (2004). Do positive interactions increase with abiotic stress? A test from a semi‐arid steppe. Proceedings of National Academy Science USA, 271(5). 10.1098/rsbl.2004.0181 PMC1810063 15504009

[ece38878-bib-0055] Maitner, B. S. , Boyle, B. , Casler, N. , Condit, R. , Donoghue, J. , Durán, S. M. , Guaderrama, D. , Hinchliff, C. E. , Jørgensen, P. M. , Kraft, N. J. , & McGill, B. (2018). The BIEN R package: A tool to access the Botanial Information and Ecology Network (BIEN) database. Methods in Ecology and Evolution, 9(2), 373–379.

[ece38878-bib-0056] Meng, X. , Huang, Z. , Di, J. , Mu, D. , Wang, Y. , Zhao, X. , Zhao, H. , Zhu, W. , Li, X. , Kong, L. , & Xing, L. (2015). Expression of human epidermal growth factor receptor‐2 in resected rectal cancer. Medicine, 94(47), 1–8. 10.1097/MD.0000000000002106 PMC5058996 26632727

[ece38878-bib-0112] Michalet, R. , Brooker, R. W. , Cavieres, L. A. , Kikvidze, Z. , Lortie, C. J. , Pugnaire, F. I. , Valiente‐Banuet, A. , & Callaway, R. M. (2006). Do biotic interactions shape both sides of the humped‐back model of species richness in plant communities? Ecology Letters, 9(7), 767–773. 16796565 10.1111/j.1461-0248.2006.00935.x

[ece38878-bib-0057] Moles, A. T. , Warton, D. I. , Warman, L. , Swenson, N. G. , Laffan, S. W. , Zanne, A. E. , Pitman, A. , Hemmings, F. A. , & Leishman, M. R. (2009). Global patterns in plant height. Journal of Ecology, 97(5), 923–932. 10.1111/j.1365-2745.2009.01526.x

[ece38878-bib-0058] Mucina, L. , Tsakalos, J. L. , & Macintyre, P. D. (2019). Ecology, biodiversity and mining: science and solving the challenges. In A. B. Fourie & M. Tibbett (Eds.), Mine Closure 2019: Proceedings of the 13th International Conference on Mine Closure (pp. 19–34). Australian Centre for Geomechanics.

[ece38878-bib-0059] Nakata, P. A. (2015). An assessment of engineered calcium oxalate crystal formation on plant growth and development as a step toward evaluating its use to enhance plant defense. PLoS One, 10(10), e0141982. 26517544 10.1371/journal.pone.0141982 PMC4627732

[ece38878-bib-0061] Navarro‐Cano, J. A. , Goberna, M. , Valiente‐Banuet, A. , Montesinos‐Navarro, A. , Garcia, C. , & Verdú, M. (2014). Plant phylodiversity enhances soil microbial productivity in facilitation‐driven communities. Oecologia, 174, 909–920. 10.1007/s00442-013-2822-5 24233688

[ece38878-bib-0062] Navarro‐Cano, J. , Verdu, M. , & Goberna, M. (2017). Trait‐based selection of nurse plants to restore ecosystem functions in mine tailings. Journal of Applied Ecology, 1–12.29749182

[ece38878-bib-0063] Oksanen, J. , Blanchet, F. G. , Friendly, M. , Kindt, R. , Legendre, P. , McGlinn, D. , Minchin, P. R. , O’Hara, R. B. , Simpson, G. L. , Solymos, P. , Stevens, M. H. H. , Szoecs, E. , & Wagner, H. (2016). vegan: Community Ecology Package. R package version 2.4‐1. https://CRAN.R‐project.org/package=vegan

[ece38878-bib-0104] Ostertag, R. , Inman‐Narahari, F. , Cordell, S. , & Giardina, C. P. (2014). Forest structure in low‐diversity tropical forests: A study of Hawaiian wet and dry forests. PLoS One, 9(8), e103268. 25162731 10.1371/journal.pone.0103268 PMC4146472

[ece38878-bib-0065] Padilla, F. M. , & Pugnaire, F. I. (2006). The role of nurse plants in the restoration of degraded environments. Frontiers in Ecology and Environment, 4(4), 196–202.

[ece38878-bib-0066] Palmer, M. A. , Bernhardt, E. S. , Schlesinger, W. H. , Eshleman, K. N. , Foufoula‐Georgiou, E. , Hendryx, M. S. , Hendryx, M. S. , Lemly, A. D. , Likns, G. E. , Loucks, O. L. , Power, M. E. , White, P. S. , & Wilcock, P. R. (2010). Mountaintop mining consequences. Science, 327, 148–149. 20056876 10.1126/science.1180543

[ece38878-bib-0098] Paniw, M. , Salguero‐Gómez, R. , & Ojeda, F. (2017). Transient facilitation of resprouting shrubs in fire‐prone habitats. Journal of Plant Ecology, 11(3), 475–483.

[ece38878-bib-0067] Pashirzad, M. , Ejtehadi, H. , Vaezi, J. , & Shefferson, R. P. (2019). Plant‐plant interactions influence phylogenetic diversity at multiple spatial scales in a semi‐arid mountain rangeland. Oecologia, 189, 1–11. 10.1007/s00442-019-04345-9 30783773

[ece38878-bib-0068] Perring, M. P. , Standish, R. J. , Price, J. N. , Craig, M. D. , Erickson, T. E. , Ruthrof, K. X. , Whiteley, A. S. , Valentine, L. E. , & Hobbs, R. J. (2015). Advances in restoration ecology: rising to the challenges of the coming decades. Ecosphere, 6, 131. 10.1890/ES15-00121.1

[ece38878-bib-0069] Pistón, N. , Schob, C. , Armas, C. , & Prieto, I. (2016). Contribution of co‐occurring shrub species to community richness and phylogenetic diversity along an environmental gradient. Perspectives in Plant Ecology, Evolution and Systematics, 19, 30–39. 10.1016/j.ppees.2016.02.002

[ece38878-bib-0070] Pitz, C. , Mahy, G. , Vermeulen, C. , Marlet, C. , & Séleck, M. (2015). Developing biodiversity indicators on a stakeholder’s opinions basis: the gypsum industry key performance indicators framework. Environmental Science and Pollution Reserve. 10.1007/s11356-015-5269-x 26347416

[ece38878-bib-0071] Prach, K. , & Tolvanen, A. (2016). How can we restore biodiversity and ecosystem services in mining and industrial sites? Environmental Science and Pollution Reserve, 23, 13587–13590. 10.1007/s11356-016-7113-3 27376366

[ece38878-bib-0072] Pratiwi , Narendra, B. H. , Siregar, C. A. , Turjaman, M. , Hidayat, A. , Rachmat, H. H. , Mulyanto, B. , Suwardi , Iskandar , Maharani, R. , Rayadin, Y. , Prayudyaningsih, R. , Yuwati, T. W. , Prematuri, R. , & Susilowati, A. (2021). Managing and reforesting degraded post‐mining landscape in Indonesia: A review. Landscape, 10, 65. 10.3390/land10060658

[ece38878-bib-0073] Qian, H. , & Jin, Y. (2016). An updated megaphylogeny of plants, a tool for generating plant phylogenies and an analysis of phylogenetic community structure. Journal of Plant Ecology, 9, 233–239. 10.1093/jpe/rtv047

[ece38878-bib-0074] Rahmanian, S. , Ejtehadi, H. , Farzam, M. , Hejda, M. , Memariani, F. , & Pyšek, P. (2021). Does the intensive grazing and aridity change the relations between the dominant shrub *Artemisia kopetdaghensis* and plants under its canopies? Ecology and Evolution, 11(20), 14115–14124. 34707844 10.1002/ece3.8124 PMC8525166

[ece38878-bib-0075] Rahmanian, S. , Hejda, M. , Ejtehadi, H. , Farzam, M. , Memariani, F. , & Pyšek, P. (2019). Effects of livestock grazing on soil, plant functional diversity, and ecological traits vary between regions with different climates in northeastern Iran. Ecology and Evolution, 9(14), 8225–8237. 10.1002/ece3.5396 31380085 PMC6662393

[ece38878-bib-0076] Ren, H. , Yang, L. , & Liu, N. (2008). Nurse plant theory and its application in ecological restoration in lower subtropics in China. Progress in Natural Science, 18, 137–142.

[ece38878-bib-0077] Rey Benayas, J. M. , Bullock, J. M. , & Newton, A. C. (2008). Creating woodland islets to reconcile ecological restoration, conservation, and agricultural land use. Frontiers in Ecology and Environment, 6, 329–336. 10.1890/070057

[ece38878-bib-0078] Sandell Festin, E. , Tigabu, M. , Chileshe, M. N. , Syampungani, S. , & Odén, P. C. (2019). Progresses in restoration of post‐mining landscape in Africa. Journal of Forestry Research, 30, 381–396. 10.1007/s11676-018-0621-x

[ece38878-bib-0103] Singh, K. , Pandey, V. C. , Singh, B. , & Singh, R. R. (2012). Ecological restoration of degraded sodic lands through afforestation and cropping. Ecological Engineering, 43, 70–80. 10.1016/j.ecoleng.2012.02.029

[ece38878-bib-0081] Smith, S. A. , & Brown, J. W. (2018). Constructing a broadly inclusive seed plant phylogeny. American Journal of Botany, 105, 302–314. 10.1002/ajb2.1019 29746720

[ece38878-bib-0082] Soliveres, S. , Maestre, F. T. , Bowker, M. A. , Torices, R. , Quero, J. L. , García‐Gómez, M. , Cabrera, O. , Cea, A. P. , Coaguila, D. , Eldridge, D. J. , & Espinosa, C. I. (2014). Functional traits determine plant co‐occurrence more than environment or evolutionary elatedness in global drylands. Perspectives in Plant Ecology, Evolution and Systematics, 16(4), 164–173. 10.1016/j.ppees.2014.05.001 PMC4407970 25914604

[ece38878-bib-0083] Soliveres, S. , & Maestre, F. T. (2014). Plant‐plant interactions, environmental gradients and plant diversity: A global synthesis of community‐level studies. Perspectives in Plant Ecology, Evolution and Systematics, 16(4), 154–163. 10.1016/j.ppees.2014.04.001 PMC4407974 25914603

[ece38878-bib-0084] Soliveres, S. , Smit, C. , & Maestre, F. T. (2015). Moving forward on facilitation research: response to changing environments and effects on the diversity, functioning and evolution of plant communities. Biological Reviews, 90(1), 297–313. 10.1111/brv.12110 24774563 PMC4407973

[ece38878-bib-0085] Soliveres, S. , Torices, R. , & Maestre, F. T. (2012). Environmental conditions and biotic interactions acting together promote phylogenetic randomness in semi‐arid plant communities: new methods help to avoid misleading conclusions. Journal of Vegetation Science, 23(5), 822–836. 10.1111/j.1654-1103.2012.01410.x 25983536 PMC4430811

[ece38878-bib-0106] Sonnier, G. P. , McAlister, L. , & Rutz, O. (2012). A dynamic model of the effect of online communications on firm sales. Marketing Science, 30(4), 702–716.

[ece38878-bib-0102] Swenson, N. G. , Enquist, B. J. , Pither, J. , Thompson, J. , & Zimmerman, J. K. (2006). The problem and promise of scale dependency in community phylogenetics. Ecology, 87, 2418–2424. 10.1890/0012-9658(2006)87[2418:TPAPOS]2.0.CO;2 17089650

[ece38878-bib-0087] Taft, J. B. , & Kron, Z. P. (2014). Evidence of species and functional group attrition in shrubencroached prairie: implications for restoration. The American Midllan. Natturalist, 172(2), 252–265. 10.1674/0003-0031-172.2.252

[ece38878-bib-0088] Tordof, G. M. , Baker, A. J. M. , & Willis, A. J. (2000). Current approaches to the revegetation and reclamation of metalliferous mine wastes. Chemosphere, 41, 219–228. 10.1016/S0045-6535(99)00414-2 10819204

[ece38878-bib-0089] Tucker, C. M. , Cadotte, M. W. , Carvalho, S. B. , Davies, T. J. , Ferrier, S. , Fritz, S. A. , Grenyer, R. , Helmus, M. R. , Jin, L. S. , Mooers, A. O. , Pavoine, S. , Purschke, O. , Redding, D. W. , Rosauer, D. F. , Winter, M. , & Mazel, F. (2017). A guide to phylogenetic metrics for conservation, community ecology and macroecology. Biological Review, 92, 698–715. 10.1111/brv.12252 PMC5096690 26785932

[ece38878-bib-0090] Valiente‐Banuet, A. , & Verdu, M. (2007). Facilitation can increase the phylogenetic diversity of plant communities. Ecology Letters, 10, 1029–1036. 10.1111/j.1461-0248.2007.01100.x 17714492

[ece38878-bib-0091] Valiente‐Banuet, A. , & Verdu, M. (2013). Plant facilitation and phylogenies. Annual Review in Ecology, Evolution and Systematics, 44, 347–366.

[ece38878-bib-0092] Vega‐Alvarez, J. , Garcia‐Rodriguez, J. , & Cayuela, L. (2019). Facilitation beyond specie richness. Journal of Ecology, 107(2), 722–734.

[ece38878-bib-0093] Wang, Y. , Wu, X. , He, S. , & Niu, R. (2021). Eco‐environmental assessment model of the mining area in Gongyi, China. Scientific Reports, 11, 17549. 34475428 10.1038/s41598-021-96625-9 PMC8413286

[ece38878-bib-0094] Webb, C. O. , Ackerly, D. D. , McPeek, M. A. , & Donoghue, M. J. (2002). Phylogenies and community ecology. Annual Review in Ecology, Evolution and Systematics, 33, 475–505. 10.1146/annurev.ecolsys.33.010802.150448

[ece38878-bib-0095] Wickham, H. (2011). ggplot2. Wiley Interdisciplinary Reviews: Computational Statistics, 3(2), 180–185. 10.1002/wics.147

[ece38878-bib-0096] Wilcoxon, F. (1945). Individual comparisons by ranking methods. Biometrics, 1(7), 80–83. 10.2307/3001968

[ece38878-bib-0097] Winkler, N. , Weymann, W. , Auge, H. , Klotz, S. , Finkenbein, P. , & Heilmeier, H. (2014). Drought resistance of native pioneer species indicates potential suitability for restoration of post‐mining areas. Web Ecology, 14(1), 65–74. 10.5194/we-14-65-2014

[ece38878-bib-0116] Zhenqi, H. , Peijun, W. , & Li, J. (2012). Ecological restoration of abandoned mine land in China. Journal of Resources and Ecology, 3(4), 289–296.

