# Supplementary material for: Plant–plant interactions determine natural restoration of plant biodiversity over time, in a degraded mined land
Source: Ecol Evol. 2022 Apr 30;12(5):e8878. doi: 10.1002/ece3.8878 (PMC9055295; doi:10.1002/ece3.8878)
Supplement: Supplementary file 1 — Appendix S1‐S4 [file ECE3-12-e8878-s001.docx]

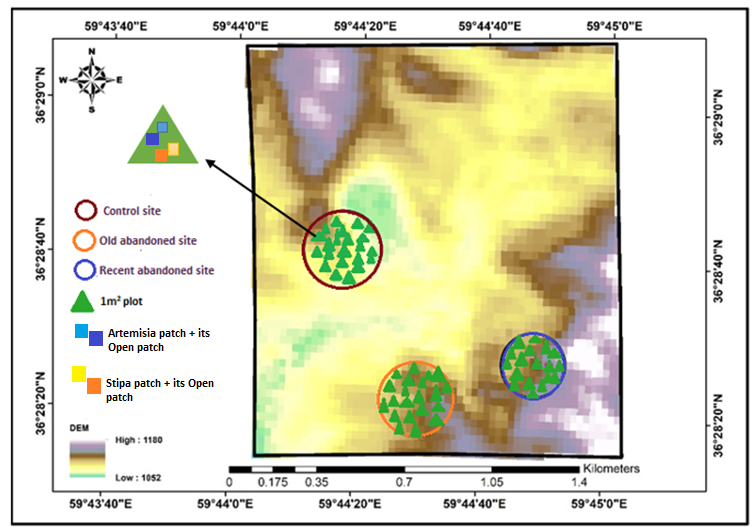


Appendix S1. The study region representing three study sites with different ages of land abandonment in Cement-Shargh located in northeastern Iran. Within each study site, 40 1 m^2^ plots were established to assess vegetation at the plot scale with respect to presence (20 plots) or absence (20 plots) of nurse species (i.e. *Artemisia sieberi* and *Stipa arabica*). In addition, in each 1m^2^ plot including nurse species, area beneath each Artemesia patch and Stipa patch was sampled using 0.5 x 0.5 m quadrats. The same number of quadrats were established adjacent to these patches to assess vegetation without nurse plant effects (i.e. open patch).

Appendix S3. Pearson correlation matrix for functional traits studied. Life form and Life span traits were transformed to ordinal values.

|  | SLA | plant height | seed mass | LDMC | C | N | life form | Life span |
| --- | --- | --- | --- | --- | --- | --- | --- | --- |
| SLA | 1 |  |  |  |  |  |  |  |
| plant height | 0.18 | 1 |  |  |  |  |  |  |
| seed mass | 0.03 | 0.12 | 1 |  |  |  |  |  |
| LDMC | -0.29 | -0.14 | 0.11 | 1 |  |  |  |  |
| C | 0.40 | 0.31 | 0.19 | 0.17 | 1 |  |  |  |
| N | 0.12 | -0.10 | 0.00 | -0.13 | -0.42 | 1 |  |  |
| life form | -0.24 | -0.02 | -0.26 | -0.07 | -0.10 | -0.39 | 1 |  |
| Life span | -0.33 | -0.01 | -0.15 | -0.05 | -0.13 | -0.45 | 0.56 | 1 |

Appendix S2. A list of Plant species presented in the sites, their mean coverage in each site and their functional trait values.

| species | mean cover age (Control site) | mean coverage  (Recent aband. site) | mean coverage (Old aband. site) | Plant species abbreviation | Life form | SLA ( m2.kg-1) | plant height (cm) | seed mass (mg) | LDMC (g) | C ( mg.g-1) | Life span | N (mg.g-1) |
| --- | --- | --- | --- | --- | --- | --- | --- | --- | --- | --- | --- | --- |
| *Rosa persica* [Michx. ex Juss.](http://www.theplantlist.org/tpl1.1/record/rjp-15223) | 0.15 | 0.40 | 0 | Ros.per | shrub | 13.07 | 22.30 | NA | 0.06 | 454.90 | Perennial | 0.001 |
| *Artemisia scoparia* [Waldst. & Kitam.](http://www.theplantlist.org/tpl1.1/record/gcc-142288) | 0.1 | 0.03 | 0.21 | Art.sco | herb | 13.85 | 39.83 | 11.14 | 0.01 | 456.44 | Biennial | 0.0019 |
| ***Prunus scoparia* (Spach) C.K.Schneid.** | 0 | 0 | 0.20 | Pru.sco | shrub | 15.02 | 44.66 | 35.60 | 0.06 | 474.22 | Perennial | 0.002 |
| *Carex stenophylla* [Wahlenb.](http://www.theplantlist.org/tpl1.1/record/kew-231141) | 0.25 | 0.75 | 0.36 | Car.ste | grass-like | 12.85 | 12.25 | 0.93 | 0.04 | 438.34 | Perennial | 0.0015 |
| *Poa bulbosa* L. | 0.51 | 0.77 | 0.62 | Poa.bul | grass | 10.25 | 26.2 | 0.90 | 0.04 | 438.213 | Perennial | 0.0012 |
| ***Stachys trinervis* Aitch. & Hemsl.** | 0.11 | 0.01 | 0.17 | Sta.tri | shrub | 29.75 | 42.56 | 2.13 | 0.05 | 451.861 | Perennial | 0.0019 |
| *Salsola kali* L. | 0.03 | 0.01 | 0.14 | Sal.kal | herb | 9.13 | 47.38 | 2.34 | 0.004 | 335.64 | Annual | 0.0032 |
| *Stipa arabica* [Trin. & Rupr.](http://www.theplantlist.org/tpl1.1/record/kew-444697) | 0.49 | 0.40 | 0.55 | Sti.ara | grass | 24.29 | 80.00 | 2.79 | 0.009 | 450.495 | Perennial | 0.0023 |
| ***Cleome coluteoides* Boiss.** | 0.11 | 0 | 0.18 | Cle.col | herb | 60.33 | 48.00 | 1.70 | 0.01 | NA | Annual | NA |
| *Bromus tectorum* L. | 0.16 | 0 | 0.39 | Bro.tec | grass | 18.55 | 11.75 | 3.23 | 0.002 | 426.05 | Annual | 0.0009 |
| *Pennisetum sp.* | 0 | 0 | 0.07 | Penn.sp | grass | 12.83 | 32.01 | 4.38 | 0.21 | 439.07 | Perennial | 0.0018 |
| *Peganum harmala* L. | 0.1 | 0.27 | 0.05 | Peg.har | herb | 16.63 | 43.70 | 2.64 | NA | 331.40 | Perennial | 0.0032 |
| *Crambe kotschyana* Boiss. | 0 | 0 | 0.02 | Cra.kot | herb | NA | 60.00 | 35.30 | 0.004 | 422.38 | Perennial | 0.0017 |
| *Centaurea virgata* Lam. | 0 | 0 | 0.12 | Cen.vir | herb | 20.77 | 29.50 | 11.04 | 0.003 | 445.06 | Perennial | 0.0019 |
| *Zygophyllum sp.* | 0.05 | 0.02 | 0.08 | Zig.sp | herb | 2.87 | 6.08 | 6.37 | NA | NA | Annual | 0.0050 |
| *Zosima absirthifolia* Link | 0 | 0 | 0.02 | Zos.abs | herb | 25.20 | 51.59 | 5.46 | 0.82 | 448.08 | Perennial | 0.0017 |
| ***Sophora pachycarpa* C.A.Mey.** | 0.03 | 0.16 | 0.09 | Sop.pac | herb | 27.47 | 33.00 | NA | 21.47 | 456.72 | Annual | 0.0029 |
| *Capparis spinosa* L. | 0.01 | 0.01 | 0.02 | Cap.spi | herb | 12.88 | 3.67 | 6.46 | 0.07 | 413.99 | Perennial | 0.0036 |
| *Aegilups sp.* | 0.04 | 0.09 | 0.05 | Aeg.sp | grass | 30.25 | 15.40 | 3.22 | 0.003 | 432.93 | Annual | 0.0011 |
| *Lactuca orientalis* [(Boiss.) Boiss.](http://www.theplantlist.org/tpl1.1/record/gcc-138369) | 0.07 | 0.10 | 0.14 | Lac.ori | herb | 30.66 | 24.33 | 1.21 | 0.003 | 435.92 | Perennial | 0.0019 |
| ***Noaea mucronata* (Forssk.) Asch. & Schweinf.** | 0.13 | 0.02 | 0.05 | Noa.muc | herb | 19.30 | 25.25 | 1.72 | 0.003 | 385.31 | Perennial | 0.0032 |
| *Erodium cicutarium* [(L.) L'Hér.](http://www.theplantlist.org/tpl1.1/record/kew-2798183) | 0.18 | 0 | 0.2 | Ero.cic | herb | 16.77 | 30.75 | 2.10 | 0.02 | 451.51 | Annual | NA |
| *Alhagi camelorum* DC. | 0.06 | 0 | 0.16 | Alh.cam | shrub | 23.47 | 38.00 | NA | 0.47 | 456.72 | Perennial | 0.0024 |
| *Cousinia smirnowii* [Trautv.](http://www.theplantlist.org/tpl1.1/record/gcc-22655) | 0.05 | 0 | 0 | Cou.smi | herb | 16.86 | 27.33 | 30.66 | 0.053 | 435.92 | Perennial | 0.0019 |
| *Echinops sp.* | 0.03 | 0.01 | 0.05 | Ech.sp | herb | 11.45 | 107.50 | 29.56 | 0.44 | 435.92 | Perennial | 0.0022 |
| *Cousinia eryngioides* Boiss. | 0.07 | 0.04 | 0.09 | Cou.ery | herb | 93.53 | 87.66 | 27.50 | 0.095 | 435.924 | Perennial | 0.0019 |
| *Euphorbia virgata* [Waldst. & Kit.](http://www.theplantlist.org/tpl1.1/record/kew-82765) | 0.03 | 0.02 | 0.08 | Eup.vir | herb | 82.75 | 8.58 | 3.20 | 0.0001 | 443.32 | Annual | 0.0029 |
| *Astragalus citrinus* Boiss. | 0.05 | 0.01 | 0.1 | Ast.cit | herb | 25.18 | 14.00 | 3.04 | 0.01 | 431.56 | Perennial | 0.0028 |
| *Geranium kotchyi* Boiss. | 0 | 0 | 0.06 | Ger.kot | herb | 30.22 | 12.00 | NA | 0.003 | 451.51 | Annual | NA |
| *Eryngium bungei* Boiss. | 0 | 0 | 0.1 | Ery.bun | herb | 16.76 | 24.33 | 1.21 | 0.003 | 448.08 | Perennial | 0.0020 |
| *Reseda lutea* L. | 0 | 0 | 0.05 | Res.lut | herb | 17.93 | 37.50 | 0.83 | 0.059 | NA | Biennial | NA |
| *Cirsium arvense* (L.)Scop. | 0 | 0 | 0.11 | Cir.arv | herb | 15.45 | 84.00 | 1.30 | 0.022 | 408.32 | Perennial | 0.0026 |
| *Pimpinella_saxifraga* L. | 0 | 0 | 0.08 | Pim.sax | herb | 18.80 | 19.00 | NA | 0.18 | 448.082 | Perennial | NA |
| *Verbascum cheirantifolium* Boiss. | 0.02 | 0.02 | 0.09 | Ver.che | herb | 33.16 | 110.50 | 0.06 | 0.24 | 489.10 | Perennial | 0.0015 |
| *Taeniatherum caput-meduse* [(L.) Nevski](http://www.theplantlist.org/tpl1.1/record/kew-446237) | 0.19 | 0.13 | 0.28 | Tae.cap | grass | 16.61 | 39.00 | 17.96 | 0.014 | 430.61 | Annual | 0.0022 |
| *Convolvulus lineatus* L. | 0.02 | 0.03 | 0.12 | Con.lin | herb | 33.16 | 7.00 | 5.60 | 0.012 | 457.67 | Perennial | 0.0018 |
| *Artemisia sieberi* Besser | 0.38 | 0.35 | 0.57 | Art.sie | shrub | 35.47 | 40.03 | 11.14 | 0.019 | 456.44 | Perennial | 0.0019 |

Appendix S4. Raw data for plant biodiversity facets to prepare the results (i.e. figures and tables included in paper).

|  | PSES.mpd | FSES.mpd | q1 | q0 | mining status | nurse type | PN |
| --- | --- | --- | --- | --- | --- | --- | --- |
| 1 | 1.167119 | 1.127981 | 2.906907 | 3 | control | grass | YES |
| 2 | 1.37846 | 0.659415 | 2.749459 | 3 | control | grass | YES |
| 3 | 0.717298 | 0.408463 | 1.979626 | 2 | control | grass | YES |
| 4 | 0.566655 | 1.399009 | 1.960132 | 2 | control | grass | YES |
| 5 | -0.62962 | -0.88724 | 1.649385 | 2 | control | grass | YES |
| 6 | 1.515276 | 1.234376 | 3.543098 | 4 | control | grass | YES |
| 7 | 1.394494 | 0.639123 | 2.749459 | 3 | control | grass | YES |
| 8 | 1.053425 | 0.435636 | 2.649351 | 3 | control | grass | YES |
| 9 | 0.594835 | 1.297313 | 1.960132 | 2 | control | grass | YES |
| 10 | 0.137419 | -0.01763 | 1.818969 | 2 | control | open-grass | NO |
| 11 | 0.373776 | 0.613715 | 1.889882 | 2 | control | open-grass | NO |
| 12 | -0.25833 | -0.68397 | 1.754765 | 2 | control | open-grass | NO |
| 13 | 0.195664 | -0.01173 | 1.818969 | 2 | control | open-grass | NO |
| 14 | 0.093773 | -0.40108 | 2.336574 | 3 | control | open-grass | NO |
| 15 | 0.765815 | 0.372876 | 2 | 2 | control | open-grass | NO |
| 16 | -1.03871 | -1.12098 | 2.293381 | 3 | recent mining | grass | YES |
| 17 | -0.71142 | -0.227 | 1.569193 | 2 | recent mining | grass | YES |
| 18 | -0.70134 | -0.8195 | 2.801973 | 4 | recent mining | grass | YES |
| 19 | -0.06808 | 0.855954 | 1.987926 | 3 | recent mining | grass | YES |
| 20 | -0.29923 | -0.90462 | 2.959998 | 3 | recent mining | grass | YES |
| 21 | 0.361218 | 0.768606 | 1.889882 | 2 | recent mining | grass | YES |
| 22 | -1.35121 | -0.81833 | 2.882067 | 3 | recent mining | grass | YES |
| 23 | -1.44991 | 0.061875 | 1.761359 | 3 | recent mining | grass | YES |
| 24 | 0.781142 | 2.372811 | 2 | 2 | recent mining | open-grass | NO |
| 25 | -0.10761 | 0.244991 | 1.754765 | 2 | recent mining | open-grass | NO |
| 26 | 0.861329 | 2.6733 | 3 | 3 | recent mining | open-grass | NO |
| 27 | -0.09304 | 0.226732 | 1.754765 | 2 | recent mining | open-grass | NO |
| 28 | 0.056522 | 1.390282 | 2.586409 | 3 | recent mining | open-grass | NO |
| 29 | -1.2978 | -0.64716 | 1.417411 | 2 | recent mining | open-grass | NO |
| 30 | -0.06529 | 1.334152 | 1.754765 | 2 | recent mining | open-grass | NO |
| 31 | -0.81194 | 0.907761 | 2.552028 | 3 | recent mining | open-grass | NO |
| 32 | -0.15808 | -0.53194 | 2.688075 | 3 | old mining | grass | YES |
| 33 | -1.03809 | -0.95578 | 2.746735 | 3 | old mining | grass | YES |
| 34 | 0.064726 | -1.23979 | 2.889098 | 3 | old mining | grass | YES |
| 35 | 0.195874 | -0.31749 | 2.828427 | 3 | old mining | grass | YES |
| 36 | -0.0934 | -0.27245 | 2.688075 | 3 | old mining | grass | YES |
| 37 | -1.82976 | -1.07662 | 2.383857 | 3 | old mining | grass | YES |
| 38 | 0.086555 | -0.85614 | 3.438876 | 4 | old mining | grass | YES |
| 39 | 0.193409 | -0.36732 | 2.828427 | 3 | old mining | grass | YES |
| 40 | 0.493963 | -0.39111 | 2.552028 | 3 | old mining | open-grass | NO |
| 41 | 1.38168 | 0.565727 | 2.828427 | 3 | old mining | open-grass | NO |
| 42 | 0.43524 | -0.21715 | 1.889882 | 2 | old mining | open-grass | NO |
| 43 | -1.94544 | 0.152144 | 1.889882 | 2 | old mining | open-grass | NO |
| 44 | 0.513412 | -0.35603 | 2.552028 | 3 | old mining | open-grass | NO |
| 45 | 0.40171 | 0.477278 | 1.889882 | 2 | old mining | open-grass | NO |
| 46 | 0.405541 | -0.17011 | 1.889882 | 2 | old mining | open-grass | NO |
| 47 | -1.95526 | 0.145317 | 1.889882 | 2 | old mining | open-grass | NO |
| 48 | 0.648077 | -0.34274 | 2.608282 | 3 | control | grass | YES |
| 49 | 1.350934 | -0.02405 | 2.749459 | 3 | control | grass | YES |
| 50 | 1.408539 | 0.30005 | 3 | 3 | control | grass | YES |
| 51 | 1.256284 | 0.127044 | 2.704797 | 3 | control | grass | YES |
| 52 | 0.22639 | -0.80483 | 2.455025 | 3 | control | grass | YES |
| 53 | 1.034318 | -0.36997 | 3.216463 | 4 | control | grass | YES |
| 54 | -0.39284 | -0.90115 | 1.649385 | 2 | control | grass | YES |
| 55 | -0.87962 | -1.10704 | 1.506993 | 2 | control | grass | YES |
| 56 | 0.344313 | -0.46885 | 1.889882 | 2 | control | open-grass | NO |
| 57 | 0.036854 | -0.75427 | 1.818969 | 2 | control | open-grass | NO |
| 58 | -0.43054 | -1.43938 | 1.889882 | 2 | control | open-grass | NO |
| 59 | 1.302758 | -0.30712 | 2.828427 | 3 | control | open-grass | NO |
| 60 | 0.738701 | -0.36978 | 2 | 2 | control | open-grass | NO |
| 61 | 0.095379 | 0.442235 | 2.086779 | 3 | old mining | grass | YES |
| 62 | 0.34784 | 0.68493 | 1.889882 | 2 | old mining | grass | YES |
| 63 | 0.288513 | 0.160961 | 1.889882 | 2 | old mining | grass | YES |
| 64 | 0.436411 | 2.070495 | 2 | 2 | old mining | grass | YES |
| 65 | 1.360034 | 1.683753 | 2.749459 | 3 | old mining | grass | YES |
| 66 | 0.834801 | -0.06485 | 2.729839 | 3 | old mining | grass | YES |
| 67 | 0.374721 | 0.95552 | 2 | 2 | old mining | open-grass | NO |
| 68 | 0.439149 | -0.24914 | 2 | 2 | old mining | open-grass | NO |
| 69 | 0.373622 | 2.584306 | 1.889882 | 2 | old mining | open-grass | NO |
| 70 | 0.756317 | 0.227186 | 2 | 2 | recent mining | grass | YES |
| 71 | 0.327062 | 0.029472 | 1.889882 | 2 | recent mining | grass | YES |
| 72 | 0.37082 | 1.162359 | 1.889882 | 2 | recent mining | grass | YES |
| 73 | 0.459688 | 2.186408 | 2 | 2 | recent mining | grass | YES |
| 74 | 0.004657 | 0.174125 | 1.889882 | 2 | recent mining | grass | YES |
| 75 | 0.132694 | 0.286115 | 1.889882 | 2 | recent mining | grass | YES |
| 76 | 0.125247 | -1.29807 | 1.889882 | 2 | recent mining | open-grass | NO |
| 77 | 0.071437 | 1.115966 | 2.828427 | 3 | recent mining | open-grass | NO |
| 78 | 0.06581 | -0.109 | 1.889882 | 2 | recent mining | open-grass | NO |
| 79 | 0.326233 | -0.64442 | 2 | 2 | recent mining | open-grass | NO |
| 80 | 0.197646 | -0.80091 | 2.401289 | 3 | control | grass | YES |
| 81 | 0.41762 | -0.56488 | 2.552028 | 3 | control | grass | YES |
| 82 | -0.33936 | -1.09862 | 1.946978 | 2 | control | grass | YES |
| 83 | -0.25805 | -1.29286 | 1.960132 | 2 | control | grass | YES |
| 84 | -1.30016 | -0.89611 | 1.417411 | 2 | control | grass | YES |
| 85 | 0.441346 | -0.58174 | 2.552028 | 3 | control | grass | YES |
| 86 | -1.08482 | -0.73387 | 1.649385 | 2 | control | grass | YES |
| 87 | -0.22594 | -0.73926 | 2.311501 | 3 | control | grass | YES |
| 88 | -0.27678 | -1.36838 | 1.991741 | 2 | control | open-grass | NO |
| 89 | -1.15784 | -1.46476 | 1.649385 | 2 | control | open-grass | NO |
| 90 | -0.66953 | 0.145882 | 1.569193 | 2 | control | open-grass | NO |
| 91 | -0.51345 | 0.157956 | 1.649385 | 2 | control | open-grass | NO |
| 92 | -0.82781 | -1.42634 | 1.754765 | 2 | control | open-grass | NO |
| 93 | 0.405255 | 1.225366 | 1.889882 | 2 | control | open-grass | NO |
| 94 | -0.38491 | 0.160301 | 1.649385 | 2 | control | open-grass | NO |
| 95 | 0.252213 | -0.60417 | 3.278598 | 4 | old mining | grass | YES |
| 96 | -0.21936 | -0.97541 | 2.888846 | 3 | old mining | grass | YES |
| 97 | 0.297129 | 0.158786 | 3.278598 | 4 | old mining | grass | YES |
| 98 | -0.08004 | -0.09568 | 2.888846 | 3 | old mining | grass | YES |
| 99 | 0.310416 | -0.03018 | 3.137026 | 4 | old mining | grass | YES |
| 100 | -1.73176 | 0.080919 | 2.941713 | 3 | old mining | grass | YES |
| 101 | 0.091681 | 1.081891 | 1.889882 | 2 | old mining | open-grass | NO |
| 102 | 1.558044 | 0.491375 | 2.871746 | 3 | old mining | open-grass | NO |
| 103 | 0.384677 | 1.435631 | 2 | 2 | old mining | open-grass | NO |
| 104 | 1.452693 | 0.341816 | 2.871746 | 3 | old mining | open-grass | NO |
| 105 | 0.143553 | 1.093876 | 1.889882 | 2 | old mining | open-grass | NO |
| 106 | 1.47159 | 0.330449 | 2.828427 | 3 | old mining | open-grass | NO |
| 107 | 0.694076 | 0.251073 | 2.95428 | 4 | recent mining | grass | YES |
| 108 | -2.09921 | -1.05431 | 1.889882 | 2 | recent mining | grass | YES |
| 109 | 1.223812 | 1.106945 | 3.389245 | 4 | recent mining | grass | YES |
| 110 | -0.55791 | -1.3668 | 1.889882 | 2 | recent mining | grass | YES |
| 111 | 0.776998 | 1.239146 | 2.971004 | 4 | recent mining | grass | YES |
| 112 | -0.53898 | -1.0638 | 1.889882 | 2 | recent mining | grass | YES |
| 113 | 1.278001 | 0.805804 | 3.361253 | 4 | recent mining | grass | YES |
| 114 | 0.380629 | 0.692207 | 1.889882 | 2 | recent mining | grass | YES |
| 115 | 0.696815 | 1.37269 | 2 | 2 | recent mining | open-grass | NO |
| 116 | -0.73401 | -0.28772 | 1.569193 | 2 | recent mining | open-grass | NO |
| 117 | -0.37705 | -1.40948 | 1.960132 | 2 | recent mining | open-grass | NO |
| 118 | -2.14817 | -0.90588 | 1.569193 | 2 | recent mining | open-grass | NO |
| 119 | 0.700786 | 1.29182 | 2 | 2 | recent mining | open-grass | NO |
| 120 | -0.01909 | -0.21935 | 2.217347 | 3 | recent mining | open-grass | NO |
| 121 | -2.17778 | -0.8451 | 1.569193 | 2 | recent mining | open-grass | NO |
| 122 | 1.458278 | 1.267439 | 2.941713 | 3 | control | shrub | YES |
| 123 | 0.616563 | 0.570694 | 2.586409 | 3 | control | shrub | YES |
| 124 | 0.789621 | -0.32337 | 2.749459 | 3 | control | shrub | YES |
| 125 | 1.066993 | 0.153561 | 3 | 3 | control | shrub | YES |
| 126 | 1.369591 | 1.154003 | 3.931112 | 4 | control | shrub | YES |
| 127 | 0.609868 | 0.554357 | 2.586409 | 3 | control | shrub | YES |
| 128 | 0.72691 | -0.24924 | 2.749459 | 3 | control | shrub | YES |
| 129 | 1.151873 | 0.239367 | 3 | 3 | control | shrub | YES |
| 130 | 0.715321 | 0.983286 | 2 | 2 | control | open-shrub | NO |
| 131 | 0.754603 | 1.453695 | 2 | 2 | control | open-shrub | NO |
| 132 | 0.439412 | 0.969447 | 1.889882 | 2 | control | open-shrub | NO |
| 133 | 1.69906 | 2.891492 | 3 | 3 | control | open-shrub | NO |
| 134 | 1.707389 | 2.897558 | 3 | 3 | control | open-shrub | NO |
| 135 | -0.49197 | -0.34571 | 1.889882 | 2 | control | open-shrub | NO |
| 136 | 0.407628 | 0.975759 | 1.889882 | 2 | control | open-shrub | NO |
| 137 | -0.09798 | -0.27213 | 2.60049 | 3 | recent mining | shrub | YES |
| 138 | 0.379935 | -0.73437 | 2.518946 | 3 | recent mining | shrub | YES |
| 139 | 1.190642 | 0.728181 | 4.189242 | 5 | recent mining | shrub | YES |
| 140 | 0.399957 | -0.72461 | 2.518946 | 3 | recent mining | shrub | YES |
| 141 | -0.52729 | -0.30614 | 1.889882 | 2 | recent mining | shrub | YES |
| 142 | 0.423664 | -0.6962 | 2.518946 | 3 | recent mining | shrub | YES |
| 143 | -0.8358 | -1.50563 | 1.754765 | 2 | recent mining | shrub | YES |
| 144 | 0.358065 | -0.21231 | 2.518946 | 3 | recent mining | shrub | YES |
| 145 | -0.42715 | -1.40091 | 1.937819 | 2 | recent mining | open-shrub | NO |
| 146 | -0.51607 | -1.45189 | 1.889882 | 2 | recent mining | open-shrub | NO |
| 147 | -0.35801 | -1.32148 | 1.937819 | 2 | recent mining | open-shrub | NO |
| 148 | -0.51853 | -1.40224 | 1.889882 | 2 | recent mining | open-shrub | NO |
| 149 | 0.955244 | 0.316559 | 2.800094 | 3 | recent mining | open-shrub | NO |
| 150 | -0.40511 | -0.2657 | 1.960132 | 2 | recent mining | open-shrub | NO |
| 151 | -0.50537 | -1.33612 | 1.889882 | 2 | recent mining | open-shrub | NO |
| 152 | 0.865851 | 0.677798 | 5.188141 | 6 | old mining | shrub | YES |
| 153 | 0.304814 | 0.769926 | 3.72457 | 5 | old mining | shrub | YES |
| 154 | 1.750638 | 1.473478 | 3.750803 | 4 | old mining | shrub | YES |
| 155 | 0.369242 | 0.854923 | 4.188152 | 5 | old mining | shrub | YES |
| 156 | 0.423208 | 0.906863 | 2.744506 | 4 | old mining | shrub | YES |
| 157 | 1.663704 | 1.169992 | 3.388144 | 4 | old mining | shrub | YES |
| 158 | 0.908653 | 0.683198 | 5.188141 | 6 | old mining | shrub | YES |
| 159 | 0.508606 | 0.828868 | 2.744506 | 4 | old mining | shrub | YES |
| 160 | 1.921236 | 1.347457 | 4.548766 | 5 | old mining | shrub | YES |
| 161 | 1.335217 | 1.883032 | 2.984328 | 3 | old mining | open-shrub | NO |
| 162 | -0.71414 | -0.10508 | 1.883326 | 3 | old mining | open-shrub | NO |
| 163 | -0.22625 | -0.45435 | 1.716357 | 2 | old mining | open-shrub | NO |
| 164 | 0.68253 | 1.755147 | 2 | 2 | old mining | open-shrub | NO |
| 165 | -1.30364 | -0.86335 | 1.569193 | 2 | old mining | open-shrub | NO |
| 166 | -0.86967 | -0.54978 | 1.716357 | 2 | old mining | open-shrub | NO |
| 167 | 1.411898 | 1.712361 | 2.984328 | 3 | old mining | open-shrub | NO |
| 168 | -0.08563 | 0.861134 | 1.754765 | 2 | old mining | open-shrub | NO |
| 169 | -0.22013 | -0.47735 | 1.716357 | 2 | old mining | open-shrub | NO |
| 170 | -0.23513 | -1.41199 | 1.987667 | 2 | control | shrub | YES |
| 171 | -0.42586 | -1.36483 | 1.918909 | 2 | control | shrub | YES |
| 172 | -0.48296 | -1.44391 | 1.889882 | 2 | control | shrub | YES |
| 173 | -0.46222 | -1.36992 | 1.918909 | 2 | control | shrub | YES |
| 174 | -0.66563 | -1.52981 | 1.818969 | 2 | control | shrub | YES |
| 175 | -0.49871 | -1.42582 | 1.937819 | 2 | control | shrub | YES |
| 176 | -0.90532 | -1.51845 | 1.731303 | 2 | control | shrub | YES |
| 177 | -0.55223 | -1.45442 | 1.90605 | 2 | control | shrub | YES |
| 178 | -0.72589 | -1.45153 | 1.796702 | 2 | control | open-shrub | NO |
| 179 | -1.04367 | -1.5069 | 1.649385 | 2 | control | open-shrub | NO |
| 180 | -0.97173 | -1.52029 | 1.698433 | 2 | control | open-shrub | NO |
| 181 | -0.67067 | -1.46474 | 1.842023 | 2 | control | open-shrub | NO |
| 182 | 0.541317 | 0.406483 | 1.946978 | 2 | old mining | shrub | YES |
| 183 | 0.206054 | 0.569689 | 1.937819 | 2 | old mining | shrub | YES |
| 184 | -1.94599 | -0.23012 | 2.749459 | 3 | old mining | shrub | YES |
| 185 | -1.9592 | -0.14086 | 2 | 2 | old mining | shrub | YES |
| 186 | 1.226733 | 1.214452 | 2.800094 | 3 | old mining | shrub | YES |
| 187 | -1.8559 | -0.51123 | 2.62469 | 3 | old mining | shrub | YES |
| 188 | -0.40993 | -0.33288 | 2.906907 | 3 | old mining | shrub | YES |
| 189 | 0.464115 | 2.300727 | 2 | 2 | old mining | open-shrub | NO |
| 190 | -0.10691 | -0.13777 | 1.754765 | 2 | old mining | open-shrub | NO |
| 191 | -0.0787 | 1.855141 | 1.754765 | 2 | old mining | open-shrub | NO |
| 192 | 0.13743 | 1.698539 | 1.889882 | 2 | old mining | open-shrub | NO |
| 193 | 0.634541 | -0.08671 | 1.960132 | 2 | recent mining | shrub | YES |
| 194 | 0.522354 | 0.685793 | 2.828427 | 3 | recent mining | shrub | YES |
| 195 | -1.87279 | -0.52688 | 2.454556 | 3 | recent mining | shrub | YES |
| 196 | -1.77323 | -0.15296 | 2.784112 | 3 | recent mining | shrub | YES |
| 197 | 1.049834 | 0.97691 | 3.709079 | 4 | recent mining | shrub | YES |
| 198 | 0.192894 | -0.23634 | 2.136004 | 3 | recent mining | shrub | YES |
| 199 | 0.943714 | 0.564321 | 2.586409 | 3 | recent mining | shrub | YES |
| 200 | 0.417733 | -1.04396 | 2 | 2 | recent mining | shrub | YES |
| 201 | -2.05397 | -1.01552 | 1.979626 | 2 | recent mining | open-shrub | NO |
| 202 | 0.459894 | 0.192471 | 2 | 2 | recent mining | open-shrub | NO |
| 203 | -0.92497 | -0.83689 | 1.506993 | 2 | recent mining | open-shrub | NO |
| 204 | -0.21096 | -1.33233 | 1.998811 | 2 | control | shrub | YES |
| 205 | 0.037763 | -0.69446 | 2.672696 | 4 | control | shrub | YES |
| 206 | -0.21519 | -0.66185 | 3.49438 | 4 | control | shrub | YES |
| 207 | -0.31377 | -1.37107 | 1.968938 | 2 | control | shrub | YES |
| 208 | 0.734908 | -0.41036 | 3.795196 | 4 | control | shrub | YES |
| 209 | -0.99562 | -1.4942 | 1.673058 | 2 | control | shrub | YES |
| 210 | -0.73089 | -1.47985 | 1.796702 | 2 | control | open-shrub | NO |
| 211 | -0.31803 | -1.35612 | 1.991741 | 2 | control | open-shrub | NO |
| 212 | -0.66737 | -1.40921 | 1.818969 | 2 | control | open-shrub | NO |
| 213 | 0.439394 | 1.503049 | 2 | 2 | control | open-shrub | NO |
| 214 | -1.79832 | -0.29351 | 2.422747 | 4 | old mining | shrub | YES |
| 215 | -0.20299 | -0.56094 | 2.702456 | 5 | old mining | shrub | YES |
| 216 | 1.406267 | -0.04652 | 3.560952 | 4 | old mining | shrub | YES |
| 217 | -0.9611 | -0.83106 | 1.457569 | 2 | old mining | shrub | YES |
| 218 | -0.91606 | -0.0166 | 3.582779 | 5 | old mining | shrub | YES |
| 219 | -0.1065 | -0.32786 | 2.702456 | 5 | old mining | shrub | YES |
| 220 | 1.38275 | 0.302582 | 3.560952 | 4 | old mining | shrub | YES |
| 221 | -1.01193 | -0.81045 | 1.457569 | 2 | old mining | shrub | YES |
| 222 | -1.36847 | -0.92412 | 1.356131 | 2 | old mining | open-shrub | NO |
| 223 | 0.100555 | -0.91518 | 2.33116 | 3 | old mining | open-shrub | NO |
| 224 | 0.713982 | 0.439514 | 2 | 2 | old mining | open-shrub | NO |
| 225 | -1.71926 | -1.19179 | 2.008098 | 3 | old mining | open-shrub | NO |
| 226 | -0.23712 | -0.13246 | 1.716357 | 2 | old mining | open-shrub | NO |
| 227 | -0.20178 | -0.82284 | 2.137309 | 3 | old mining | open-shrub | NO |
| 228 | 0.703926 | 0.427703 | 2 | 2 | old mining | open-shrub | NO |
| 229 | -1.91632 | -1.06304 | 1.968082 | 4 | old mining | open-shrub | NO |
| 230 | 0.951067 | -0.56819 | 3.626443 | 4 | recent mining | shrub | YES |
| 231 | 1.40978 | 2.010781 | 3.464102 | 4 | recent mining | shrub | YES |
| 232 | 1.634695 | 0.800949 | 4.339391 | 5 | recent mining | shrub | YES |
| 233 | 1.69073 | 2.585235 | 4.129141 | 5 | recent mining | shrub | YES |
| 234 | 1.276096 | 0.173072 | 3.389245 | 4 | recent mining | shrub | YES |
| 235 | 1.394407 | 2.338339 | 3.464102 | 4 | recent mining | shrub | YES |
| 236 | -0.70314 | -1.41538 | 1.818969 | 2 | recent mining | open-shrub | NO |
| 237 | 1.366762 | 1.452971 | 2.828427 | 3 | recent mining | open-shrub | NO |
| 238 | -0.73272 | -0.44817 | 1.818969 | 2 | recent mining | open-shrub | NO |
| 239 | 1.521251 | 1.495678 | 2.828427 | 3 | recent mining | open-shrub | NO |
| 240 | -0.6742 | -1.50524 | 1.818969 | 2 | recent mining | open-shrub | NO |
| 241 | 1.807042 | 2.606224 | 3.789291 | 4 | recent mining | open-shrub | NO |
